# Novel Scalar-on-matrix Regression for Unbalanced Feature Matrices

**DOI:** 10.1007/s12561-025-09476-7

**Published:** 2025-03-05

**Authors:** Jeremy Rubin, Fan Fan, Laura Barisoni, Andrew R. Janowczyk, Jarcy Zee

**Affiliations:** 1 Department of Biostatistics, Epidemiology, and Informatics, University of Pennsylvania Perelman School of Medicine, 210 Blockley Hall, 423 Guardian Drive, Philadelphia, PA 19104 USA; 2 Department of Biomedical Engineering, Emory University and Georgia Institute of Technology, Atlanta, GA USA; 3 Division of AI and Computational Pathology, Department of Pathology, Duke University, Durham, NC USA; 4 Division of Nephrology, Department of Medicine, Duke University, Durham, NC USA; 5 Oncology and Pathology Departments, Geneva University Hospitals, Geneva, Switzerland; 6 The Children’s Hospital of Philadelphia Research Institute, Philadelphia, PA USA

**Keywords:** Computational pathology, Image features, Scalar-on-matrix regression, Unsupervised clustering, Variable selection

## Abstract

Image features that characterize tubules from digitized kidney biopsies may offer insight into disease prognosis as novel biomarkers. For each subject, we can construct a matrix whose entries are a common set of image features (e.g., area, orientation, eccentricity) that are measured for each tubule from that subject’s biopsy. Previous scalar-on-matrix regression approaches which can predict scalar outcomes using image feature matrices cannot handle varying numbers of tubules across subjects. We propose the CLUstering Structured laSSO (CLUSSO), a novel scalar-on-matrix regression technique that allows for unbalanced numbers of tubules, to predict scalar outcomes from the image feature matrices. Through classifying tubules into one of two different clusters, CLUSSO averages and weights tubular feature values within-subject and within-cluster to create balanced feature matrices that can then be used with structured lasso regression. We develop the theoretical large tubule sample properties for the error bounds of the feature coefficient estimates. Simulation study results indicate that CLUSSO often achieves a lower false positive rate and higher true positive rate for identifying the image features which truly affect outcomes relative to a naive method that averages feature values across all tubules. Additionally, we find that CLUSSO has lower bias and can predict outcomes with a competitive accuracy to the naïve approach. Finally, we applied CLUSSO to tubular image features from kidney biopsies of glomerular disease subjects from the Nephrotic Syndrome Study Network (NEPTUNE) to predict kidney function and used subjects from the Cure Glomerulonephropathy (CureGN) study as an external validation set.

**Supplementary Information:**

The online version contains supplementary material available at 10.1007/s12561-025-09476-7.

## Introduction

### Motivation

For diagnosis of kidney disease, renal biopsies are the current gold standard. However, visual assessment of renal biopsies is subjective, time-consuming, often limited to basic qualitative descriptions, and has poor intra- and inter-reader reproducibility. Digital pathology enables characterization of biopsy tissue on a large scale, including deep learning segmentation algorithms that can detect individual objects (e.g., glomeruli, tubules, vessels) from whole slide images (WSIs) [12]. From each of these objects, computer-generated image features can comprehensively and reproducibly describe morphology, texture, and topology. These image features thus provide quantitative, granular measurements of visual and subvisual characteristics, offering the opportunity for novel scientific discoveries that are not possible using traditional pathology approaches [1].

These computer-extracted image features are hierarchical in nature, as each feature is measured for every identified object, and every subject has a unique set of identified objects. Each subject’s features can then be represented by a matrix of the form $\left\{{{\boldsymbol{X}}}_{{\boldsymbol{i}}}\in {\mathbb{R}}^{{p}_{i}\times q}: \quad i=1,\ldots ,n\right\},$ where ${p}_{i}$ is the number of identified objects for subject $i$ and $q$ is the number of features. The number of rows (i.e., objects) can vary across subjects, but the number of features is constant across subjects. Our goal is to use these feature matrices to perform regression and predict scalar outcomes ${y}_{i}\in {\mathbb{R}}.$ We ultimately aim to understand which image features are most useful for predicting outcomes to facilitate the discovery of novel image feature biomarkers.

A naïve approach to analyze these data is to average feature values across all objects within a subject such that each subject’s features are represented by a vector and standard regression techniques can be employed. However, this strategy may result in large information loss given the potentially large heterogeneity across objects. For example, prior literature has shown that the percentage of tubules in a renal biopsy with tubular atrophy is associated with worse kidney function in glomerular disease [17]. Since atrophic (diseased) tubules have thickened tubular basement membranes compared with non-atrophic (normal, non-diseased) tubules [15], averaging tubular basement membrane thickness values across all tubules ignores the expected bimodal distribution of the feature. Alternatively, we can consider scalar-on-matrix-regression methods for analysis, but existing methods all require a balanced design, i.e., matrix-valued predictors that have the same number of rows and columns across subjects. Developing a technique that better preserves the heterogeneity of the tubules in atrophic vs. non-atrophic groups may lead to better prediction of kidney function outcomes.

### Existing Scalar-on-matrix Regression Approaches

A simple approach to handle data in a scalar-on-matrix regression setting is to vectorize each subject’s matrix-valued predictor ${{\boldsymbol{X}}}_{{\boldsymbol{i}}}.$ Then, standard linear regression methods can be performed on these transformed data. A major disadvantage of this approach is that interpretations at the row and column level of the original ${{\boldsymbol{X}}}_{{\boldsymbol{i}}}$ matrix are no longer feasible, and we lose significant information about the structure of the feature matrix. In particular, we no longer retain information about differences in tubular image features between atrophic and non-atrophic tubules. Additionally, the number of parameters that needs to be estimated when vectorizing the ${{\boldsymbol{X}}}_{{\boldsymbol{i}}}$ matrix greatly increases [14].

In the past decade, several novel approaches have been proposed to handle scalar-on-matrix regression, such that the structure of the matrix-valued predictor ${{\boldsymbol{X}}}_{{\boldsymbol{i}}}$ is preserved in the model-building process (see [4, 10, 13, 14, 24–27], and [29] for examples). Zhou and Li [29] use a trace regression approach by regressing ${y}_{i}$ on $tr({{\boldsymbol{X}}}_{{\boldsymbol{i}}}{{\boldsymbol{B}}}^{{\boldsymbol{T}}}),$ where ${\boldsymbol{B}}$ is a coefficient matrix the same size as ${{\boldsymbol{X}}}_{{\boldsymbol{i}}}\in {\mathbb{R}}^{p\times q}.$ However, trace regression methods are not suitable for our setting since these models estimate coefficients for each individual matrix element rather than at the row or column level.

In contrast, Zhao and Leng [27] regress ${y}_{i}$ on ${\boldsymbol{\alpha }}^{{\boldsymbol{T}}}{{\boldsymbol{X}}}_{{\boldsymbol{i}}}{\boldsymbol{\beta}},$ where the vectors $\boldsymbol{\alpha }$ and ${\boldsymbol{\beta}}$ represent the row and column effects, respectively, of ${{\boldsymbol{X}}}_{{\boldsymbol{i}}}$ on ${y}_{i}.$ However, a limitation of this structured lasso approach of Zhao and Leng [27] is that it assumes a balanced design for the feature matrix ${{\boldsymbol{X}}}_{{\boldsymbol{i}}}$ across subjects. We will therefore develop a novel scalar-on-matrix regression model that allows for feature matrices of different sizes.

The remainder of this paper is organized in the following manner. In Sect. 2, we develop the Full Information Structured Lasso, an oracle estimator of the tubular image feature-level effects on the outcome that could be used if all tubules and tubule subgroups were observed. In Sect. 3, we describe our proposed estimator, the CLUstering Structured lasSO (CLUSSO), to be used in real data applications, where a sample of tubules are observed and tubular subgroups are unknown. We compare the performance of the Full Information Structured Lasso, CLUSSO, and a naïve strategy in a simulation study in Sect. 4. Then, we illustrate the use of CLUSSO with a real data analysis in which the CLUSSO model is trained on a cohort of subjects with glomerular disease from the Nephrotic Syndrome Study Network (NEPTUNE) to predict kidney function and is validated on subjects with glomerular disease from the Cure Glomerulonephropathy (CureGN) study in Sect. 5. Finally, we provide some concluding remarks about the utility of CLUSSO and future extensions in Sect. 6.

## Full Information Structured Lasso

### Feature Matrices, Outcomes, and Coefficient Estimation

For our application of identifying features measured at the tubule level, there are two major challenges of the observed feature matrix ${{\boldsymbol{X}}}_{{\boldsymbol{i}}}$ , which make the current scalar-on-matrix approaches infeasible without some modifications. First, the matrices ${{\boldsymbol{X}}}_{{\boldsymbol{i}}}$ have different numbers of rows for each subject, as the number of tubules per subject can vary, and we need consistent dimensions across ${{\boldsymbol{X}}}_{{\boldsymbol{i}}}$ for our sample to be modeled with an existing scalar-on-matrix regression method. Second, there is no consistent and meaningful ordering of tubules, or the rows of ${{\boldsymbol{X}}}_{{\boldsymbol{i}}},$ across subjects. Therefore, it will be crucial to transform our observed feature matrices to create a balanced and consistent row ordering across the matrices to allow for reliable estimation of coefficient estimates.

If there is heterogeneity in the tubular characteristics, then the naïve method of averaging the image features across all tubules will incur a large loss in information. In contrast, grouping the tubules into homogeneous subgroups (i.e., atrophic vs. non-atrophic) and averaging the image features within these two subgroups preserves information.

We assume that the observed tubular feature vectors are drawn from a two-component mixture of latent distributions. Suppose further that for each subject $i,$ we have their entire population of tubules, and we know which tubules belong to each of $G=2$ subgroups. These subgroups could correspond to known tubular phenotypes (e.g., atrophic vs. non-atrophic), or they could represent some other unknown subgroups representing similarity of tubular features. Within each subgroup $k,$ we can calculate the subject-level average of feature values. We call such a matrix ${{\boldsymbol{X}}}_{{\boldsymbol{i}}}^{{\ast}}\in {\mathbb{R}}^{2\times q},$ for which each row $k$ contains subject $i$’s tubular feature averages for all of their tubules in the *k*th subgroup.

The feature matrices ${{\boldsymbol{X}}}_{{\boldsymbol{i}}}^{{\ast}}$ alone do not contain information about the relative frequencies of tubules in each of the two subgroups per subject. The more tubules a subject has in a given subgroup, the more informative the corresponding subgroup-averaged image features should be in predicting the clinical outcome. For example, a subject with a large proportion of atrophic tubules would be expected to have worse kidney function than a subject with a small proportion of atrophic tubules. Therefore, for each subject and each cluster, we weigh the average of feature values for subject $i$ and subgroup $k$ by ${w}_{k}^{i},$ which represents the true proportion of tubules in cluster $k$ for subject $i.$ Then, the final design matrices ${{\boldsymbol{X}}}_{{\boldsymbol{i}}}^{{{\ast\ast}}}$ to be used for the Full Information Structured Lasso are as follows:${{\boldsymbol{X}}}_{{\boldsymbol{i}}}^{{{\ast\ast}}}=diag\left({w}_{1}^{i},{w}_{2}^{i}\right){{\boldsymbol{X}}}_{{\boldsymbol{i}}}^{\ast}.$

Since each tubular feature value for each subject is drawn from the same mixture distribution, the averages are also identically distributed across subjects. The ${{\boldsymbol{X}}}_{{\boldsymbol{i}}}^{\ast}$’s are therefore independently and identically distributed (i.i.d.) by independence of the tubular feature averages across subjects and each tubular feature average in the $k$th subgroup for each subject is drawn from the same latent distribution. We further assume that the weights ${w}_{k}^{i}$ across subjects are i.i.d. drawn from a common latent distribution. Therefore, the design matrices ${{\boldsymbol{X}}}_{{\boldsymbol{i}}}^{{\ast\ast}}=diag\left({w}_{1}^{i},{w}_{2}^{i}\right){{\boldsymbol{X}}}_{{\boldsymbol{i}}}^{\ast}$ are i.i.d. as well.

Let ${\boldsymbol{\alpha }}^{\ast}\in {\mathbb{R}}^{2\times 1}$ and ${{\boldsymbol{\beta}}}^{\ast}\in {\mathbb{R}}^{q\times 1}$ be coefficient vectors that describe the row-level and column-level effects of the feature matrices ${{\boldsymbol{X}}}_{{\boldsymbol{i}}}^{{\ast\ast}}.$ Assume the outcome model ${y}_{i}={\left({\boldsymbol{\alpha }}^{\ast}\right)}^{T}{{\boldsymbol{X}}}_{{\boldsymbol{i}}}^{{\ast\ast}}{{\boldsymbol{\beta}}}^{{\ast}}+{\upvarepsilon }_{i},$ where $vec\left({{\boldsymbol{X}}}_{{\boldsymbol{i}}}^{{\ast\ast}}\right)\sim N(0,{\boldsymbol{\Sigma}})$ with diagonal elements of ${\boldsymbol{\Sigma}}$ as 1, and ${\varepsilon }_{i}\sim N(0,{\sigma }^{2}).$ The assumptions on ${{\boldsymbol{X}}}_{{\boldsymbol{i}}}^{{\ast\ast}}$ can be met through standardization. Then, the sample on which we would fit a scalar-on-matrix regression model is as follows:

1 $$\left\{\left({{\boldsymbol{X}}}_{{\boldsymbol{i}}}^{{\ast\ast}},{y}_{i}\right);{{\boldsymbol{X}}}_{{\boldsymbol{i}}}^{{\ast\ast}}\in {\mathbb{R}}^{2\times q},{y}_{i}\in {\mathbb{R}};i=1,\ldots ,n\right\}.$$

We can adapt the structured lasso method of Zhao and Leng [27] to estimate ${\boldsymbol{\alpha }}^{{\ast}}$ and ${{\boldsymbol{\beta}}}^{{\ast}}$:

2 $$({\widehat{\boldsymbol{\alpha }}}_{{\boldsymbol{F}}}^{{\ast}},{\widehat{{\boldsymbol{\beta}}}}_{{\boldsymbol{F}}}^{{\ast}})=\mathrm{arg}\underset{\left({\boldsymbol{\alpha }}^{{\ast}},{{\boldsymbol{\beta}}}^{{\ast}}\right)\in \mathcal{E}}{\mathrm{min}}\frac{1}{n}{\Sigma }_{i=1}^{n}{\left({y}_{i}-{{\boldsymbol{\alpha }}^{{\ast}}}^{{\boldsymbol{T}}}{{\boldsymbol{X}}}_{{\boldsymbol{i}}}^{{\ast\ast}}{{\boldsymbol{\beta}}}^{{\ast}}\right)}^{2}+{\lambda }_{n}{\Vert {{\boldsymbol{\beta}}}^{{\ast}}\Vert }_{1}$$

for $\mathcal{E}=\{\left({\boldsymbol{\alpha }}^{{\ast}},{{\boldsymbol{\beta}}}^{{\ast}}\right):{\boldsymbol{\alpha }}^{{\ast}}\in {\mathbb{R}}^{2},{\Vert {\boldsymbol{\alpha }}^{{\ast}}\Vert }_{1}=1, {{\boldsymbol{\beta}}}^{{\ast}}\in {\mathbb{R}}^{q},sign({\beta }_{\left(1\right)}^{\ast})=1\},$ where ${\beta }_{\left(1\right)}^{\ast}$ is the largest component of ${{\boldsymbol{\beta}}}^{{\ast}}$ in magnitude, and ${\lambda }_{n}$ is the regularization parameter. Unlike Zhao and Leng [27], we do not regularize ${\boldsymbol{\alpha }}^{{\ast}},$ as we wish to use both the tubular subgroups for predicting the outcome. We call the estimator (${\widehat{\boldsymbol{\alpha }}}_{{\boldsymbol{F}}}^{{\ast}},{\widehat{{\boldsymbol{\beta}}}}_{{\boldsymbol{F}}}^{{\ast}})$ the Full Information Structured Lasso, as we are assuming complete knowledge of the tubular classification into the two latent subgroups and that the resulting tubular feature averages for subject $i$ are computed using the population of subject $i$’s tubules. This method could generate feature-level effects through estimation of ${{\boldsymbol{\beta}}}^{{\ast}}$ and perform variable selection by identifying features with nonzero coefficients through the penalty term of ${\Vert {{\boldsymbol{\beta}}}^{{\ast}}\Vert }_{1}.$ The magnitudes of the components of ${\widehat{{\boldsymbol{\beta}}}}_{{\boldsymbol{F}}}^{{\ast}}$ could be used to rank the importance of the image features, while further interpretations are complicated by issues of sign identifiability and normalization, as demonstrated by an illustrative example provided in Appendix A of the Supplementary Materials. Finally, these feature weights ${\widehat{{\boldsymbol{\beta}}}}_{{\boldsymbol{F}}}^{{\ast}}$ could be used to predict scalar outcomes for new subjects.

### Coefficient Estimation Error Bound

Let ${\Vert {\boldsymbol{\alpha }}^{{\ast}}\Vert }_{1}=1$ and $sign({\beta }_{\left(1\right)}^{\ast})=1.$ These conditions are met with appropriate scaling and standardization of ${{\boldsymbol{X}}}_{{\boldsymbol{i}}}^{{\ast\ast}},$ ${\boldsymbol{\alpha }}^{{\ast}},$ and ${{\boldsymbol{\beta}}}^{{\ast}}.$ Also, define ${\boldsymbol{\theta}}={{\boldsymbol{\beta}}}^{{\ast}}\otimes {\boldsymbol{\alpha }}^{{\ast}}$ and the set of nonzero elements of ${\boldsymbol{\theta}}$ as ${S}_{\theta }=\{l: {\theta }_{l}\ne 0\}$ with cardinality ${s}_{0}=|{S}_{\theta }|.$ By Zhao and Leng [27], such an estimator ${\widehat{{\boldsymbol{\beta}}}}_{{\boldsymbol{F}}}^{{\ast}}$ would have the following L1 estimation error upper bound:

3 $${\Vert {\widehat{{\boldsymbol{\beta}}}}_{{\boldsymbol{F}}}^{{\ast}}-{{\boldsymbol{\beta}}}^{{\ast}}\Vert }_{1}\le {B}_{0}{\lambda }_{n}{s}_{0}\left(1+\frac{2{\Vert {{\boldsymbol{\beta}}}^{{\ast}}\Vert }_{1}}{{\beta }_{\left(1\right)}^{\ast}}\right),$$

where

4 $${\lambda }_{n}=\left(1+{\delta }_{0}\right)\sigma \sqrt{2\left(1+a\right)\mathrm{log}(Gq)/n}\;\mathrm{and}\;{B}_{0}=4{\kappa }^{2}({s}_{0},3)$$

for any ${\delta }_{0}>0$ and $a>0,$ and ${\kappa }^{2}\left({s}_{0},3\right)>0$ is defined according to the structured restricted eigenvalue (RE) condition noted in Zhao and Leng [27]. This condition is a matrix-valued predictor extension of the RE conditions established by Bickel et al. [2] for the lasso. Essentially, the RE condition checks whether the true sparse regression coefficient vector can be estimated with high probability [28]. Next, we propose our estimator, CLUSSO, of the regression coefficients ${\boldsymbol{\alpha }}^{{\ast}}$ and ${{\boldsymbol{\beta}}}^{{\ast}}$ to address the fact that the classification of tubules into subgroups for subjects $i=1,\ldots ,n$ is unknown due to these subgroups being latent, and we only observe ${p}_{i}$ tubules per subject, rather than the full population of tubules for subject $i.$

## CLUstering Structured LasSO: CLUSSO

### Cluster-averaged and Weighted Tubular Image Feature Matrices

Let ${{\boldsymbol{T}}}_{{\boldsymbol{j}}}^{{\boldsymbol{i}}}$ for $j=1,\ldots ,{p}_{i}$ represent the feature vector for tubule $j$ for subject $i.$ Then, the matrix of all tubules across all subjects can be represented as follows: ${{\mathbb{T}}={({\boldsymbol{T}}}_{1}^{1}\ldots ,{{\boldsymbol{T}}}_{{{\boldsymbol{p}}}_{1}}^{1},{{\boldsymbol{T}}}_{1}^{2}\ldots ,{{\boldsymbol{T}}}_{{{\boldsymbol{p}}}_{2}}^{2},\cdots ,{{\boldsymbol{T}}}_{1}^{{\boldsymbol{n}}},\ldots ,{{\boldsymbol{T}}}_{{{\boldsymbol{p}}}_{{\boldsymbol{n}}}}^{{\boldsymbol{n}}})}^{T}.$ We aim to classify each of the ${\Sigma }_{i=1}^{n}{p}_{i}$ observations in ${\mathbb{T}}$ into one of two latent subgroups. Because latent subgroup tubular membership assignments are unknown, an unsupervised clustering method should be used to classify the tubules.

An unsupervised clustering method should be chosen based on several considerations including assumptions about the distribution of the tubular features or shape of the clusters, as well as the computational complexity of the clustering algorithm. For instance, if assuming that the tubular features are all i.i.d. from a finite mixture of Gaussian distributions as is done for GMM-based clustering is too strict for the data application, alternative clustering methods that are not model-based such as K-means clustering can be used. We refer the reader to Gao et al. [7] for comprehensive and practical guidelines of choosing between modern clustering methods.

After each tubule is assigned to one of the two subgroups, tubular image features within each subgroup within a subject can be averaged to calculate the estimated cluster-averaged feature matrices ${\widehat{{\boldsymbol{X}}}}_{{\boldsymbol{i}}}^{{\ast}}$: $\left\{{\widehat{{\boldsymbol{X}}}}_{{\boldsymbol{i}}}^{{\ast}}\in {\mathbb{R}}^{2\times q}:i=1,..,n\right\},$ where row $k$ of ${\widehat{{\boldsymbol{X}}}}_{{\boldsymbol{i}}}^{{\ast}}$ can be defined as follows:

5 $${\widehat{{\boldsymbol{X}}}}_{{\boldsymbol{i}},{\boldsymbol{k}}}^{{\ast}}=\frac{{\sum }_{j=1}^{{p}_{i}}I({{\boldsymbol{T}}}_{{\boldsymbol{j}}}^{{\boldsymbol{i}}} \in {\widehat{\mathbb{T}}}_{k}){{\boldsymbol{T}}}_{{\boldsymbol{j}}}^{{\boldsymbol{i}}}}{{\sum }_{j=1}^{{p}_{i}}I({{\boldsymbol{T}}}_{{\boldsymbol{j}}}^{{\boldsymbol{i}}} \in {\widehat{\mathbb{T}}}_{k})},$$

where ${\widehat{\mathbb{T}}}_{k}$ is the set of image feature vectors classified as belonging to subgroup/cluster $k=1, 2$ by the clustering. We see that ${\widehat{{\boldsymbol{X}}}}_{{\boldsymbol{i}},{\boldsymbol{k}}}^{{\ast}}$ is only defined for nonzero ${\sum }_{j=1}^{{p}_{i}}I({{\boldsymbol{T}}}_{{\boldsymbol{j}}}^{{\boldsymbol{i}}} \in {\widehat{\mathbb{T}}}_{k}),$ meaning that subject $i$ must have at least one tubule in cluster $k$ for ${\widehat{{\boldsymbol{X}}}}_{{\boldsymbol{i}},{\boldsymbol{k}}}^{{\ast}}$ to be defined. Therefore, subject $i$ must have one tubule in each cluster for their ${\widehat{{\boldsymbol{X}}}}_{{\boldsymbol{i}}}^{{\ast}}$ to be defined. We will estimate the row weights ${w}_{k}^{i}$ based on the clustering by defining our estimated weights as follows: ${ \widehat{w}}_{k}^{i}$=$\frac{1}{{p}_{i}}{\sum }_{j=1}^{{p}_{i}}I({{\boldsymbol{T}}}_{{\boldsymbol{j}}}^{{\boldsymbol{i}}} \in {\widehat{\mathbb{T}}}_{k}),$ the observed sample proportion of tubules clustered into each subgroup. Then, we weigh the estimated cluster-averaged image features per subject by our estimated weights ${\widehat{w}}_{k}^{i}$: ${\widehat{{\boldsymbol{X}}}}_{{\boldsymbol{i}}}^{{\ast\ast}}=diag\left({\widehat{w}}_{1}^{i},{\widehat{w}}_{2}^{i}\right){\widehat{{\boldsymbol{X}}}}_{{\boldsymbol{i}}}^{{\ast}}.$ The sample on which we will fit the structured lasso is as follows: $\{\left({\widehat{{\boldsymbol{X}}}}_{{\boldsymbol{i}}}^{{\ast\ast}},{y}_{i}\right);{\widehat{{\boldsymbol{X}}}}_{{\boldsymbol{i}}}^{{\ast\ast}}\in {\mathbb{R}}^{2\times q},{y}_{i}\in {\mathbb{R}};i=1,\ldots ,n\}.$ Finally, our CLUSSO estimator of ${\boldsymbol{\alpha }}^{{\ast}}$ and ${{\boldsymbol{\beta}}}^{{\ast}}$ is given by

6 $$\left({\widehat{\boldsymbol{\alpha }}}_{{\boldsymbol{C}}}^{{\ast}},{\widehat{{\boldsymbol{\beta}}}}_{{\boldsymbol{C}}}^{{\ast}}\right) = \mathrm{arg}\underset{\left({\boldsymbol{\alpha }}^{{\ast}},{{\boldsymbol{\beta}}}^{{\ast}}\right)\in \mathcal{E}}{\mathrm{min}}\frac{1}{n}{\Sigma }_{i=1}^{n}{\left({y}_{i}-{{\boldsymbol{\alpha }}^{{\ast}}}^{{\boldsymbol{T}}}{\widehat{{\boldsymbol{X}}}}_{{\boldsymbol{i}}}^{{\ast\ast}}{{\boldsymbol{\beta}}}^{{\ast}}\right)}^{2}+{\lambda }_{n}{\Vert {{\boldsymbol{\beta}}}^{{\ast}}\Vert }_{1}.$$

### Asymptotic Convergence of CLUSSO to the Full Information Structured Lasso

In this section, we demonstrate that under perfect clustering of tubules, the CLUSSO estimator converges to the Full Information Structured Lasso estimator. If the true latent tubular clusters were known, perfect clustering of tubules would occur if each tubule was assigned a label that corresponds to its known latent cluster.

The following results hold under the assumptions that ${\widehat{\mathbb{T}}}_{k}={\mathbb{T}}_{k},$ i.e., that the estimated tubular cluster labels equal the true tubular cluster labels and tubular feature vectors within the same subgroup for each subject are i.i.d. Under these assumptions, we have

7 $${\widehat{{\boldsymbol{X}}}}_{{\boldsymbol{i}},{\boldsymbol{k}}}^{{\ast}}=\frac{{\sum }_{j=1}^{{p}_{i}}I({{\boldsymbol{T}}}_{{\boldsymbol{j}}}^{{\boldsymbol{i}}} \in {\widehat{\mathbb{T}}}_{k}){{\boldsymbol{T}}}_{{\boldsymbol{j}}}^{{\boldsymbol{i}}}}{{\sum }_{j=1}^{{p}_{i}}I({{\boldsymbol{T}}}_{{\boldsymbol{j}}}^{{\boldsymbol{i}}} \in {\widehat{\mathbb{T}}}_{k})}=\frac{{\sum }_{j=1}^{{p}_{i}}I({{\boldsymbol{T}}}_{{\boldsymbol{j}}}^{{\boldsymbol{i}}} \in {\mathbb{T}}_{k}){{\boldsymbol{T}}}_{{\boldsymbol{j}}}^{{\boldsymbol{i}}}}{{\sum }_{j=1}^{{p}_{i}}I({{\boldsymbol{T}}}_{{\boldsymbol{j}}}^{{\boldsymbol{i}}} \in {\mathbb{T}}_{k})}.$$

Therefore, each ${\widehat{{\boldsymbol{X}}}}_{{\boldsymbol{i}},{\boldsymbol{k}}}^{{\ast}}$ is an average of ${\sum }_{j=1}^{{p}_{i}}I({{\boldsymbol{T}}}_{{\boldsymbol{j}}}^{{\boldsymbol{i}}} \in {\mathbb{T}}_{k})$ i.i.d. observations of tubular feature vectors that have been correctly identified as belonging to subgroup $k.$ Then, by the Weak Law of Large Numbers, we can assert that ${\widehat{{\boldsymbol{X}}}}_{{\boldsymbol{i}},{\boldsymbol{k}}}^{{\ast}}\stackrel{P}{\to }E\left({{\boldsymbol{T}}}^{{\boldsymbol{i}},{\boldsymbol{k}}}\right)$ as ${\sum }_{j=1}^{{p}_{i}}I({{\boldsymbol{T}}}_{{\boldsymbol{j}}}^{{\boldsymbol{i}}} \in {\mathbb{T}}_{k})\to \infty ,$ where ${{\boldsymbol{T}}}^{{\boldsymbol{i}},{\boldsymbol{k}}}$ denotes a representative tubular feature vector from cluster/subgroup $k$ for subject $i.$ Then, we have ${\widehat{{\boldsymbol{X}}}}_{{\boldsymbol{i}}}^{{\ast}}\stackrel{P}{\to }E({{\boldsymbol{T}}}^{{\boldsymbol{i}}})$ as ${\sum}_{j-1}^{{p}_{i}}I({\boldsymbol{T}}_{\boldsymbol{j}}^{\boldsymbol{i}} \in {\mathbb{T}}_{k}) \to \infty \; \forall k,$ where ${{\boldsymbol{T}}}^{{\boldsymbol{i}}}$ denotes a representative tubular feature matrix for subject $i.$ Thus, we have that ${\widehat{{\boldsymbol{X}}}}_{{\boldsymbol{i}}}^{{\ast}}\stackrel{P}{\to }{{\boldsymbol{X}}}_{{\boldsymbol{i}}}^{{\ast}},$ where ${{\boldsymbol{X}}}_{{\boldsymbol{i}}}^{{\ast}}$ is the matrix of true population averages of feature values for subject $i$ as defined in Sect. 2.1.

Note that there is also uncertainty in the estimation of the true proportions ${w}_{k}^{i}$ of tubules per subject $i$ from each cluster $k={1,2}.$ Recall from Sect. 3.1 that we estimate the weights by ${\widehat{w}}_{k}^{i}$=$\frac{1}{{p}_{i}}{\sum }_{j=1}^{{p}_{i}}I({{\boldsymbol{T}}}_{{\boldsymbol{j}}}^{{\boldsymbol{i}}} \in {\widehat{\mathbb{T}}}_{k})$ $\forall k.$ By the Weak Law of Large Numbers, ${\widehat{w}}_{1}^{i}\stackrel{P}{\to }{w}_{1}^{i}$ and ${\widehat{w}}_{2}^{i}\stackrel{P}{\to }{w}_{2}^{i}$ as ${p}_{i}\to \infty .$ Therefore, we have that $diag\left({\widehat{w}}_{1}^{i},{\widehat{w}}_{2}^{i}\right)\stackrel{P}{\to }diag\left({w}_{1}^{i},{w}_{2}^{i}\right)$ as ${p}_{i}\to \infty .$ Then, the final estimated design matrices ${\widehat{{\boldsymbol{X}}}}_{{\boldsymbol{i}}}^{{\ast\ast}}$ to be fit with the structured lasso for CLUSSO will converge asymptotically to the true ${{\boldsymbol{X}}}_{{\boldsymbol{i}}}^{{\ast\ast}}$ by the continuous mapping theorem:

8 $${\widehat{{\boldsymbol{X}}}}_{{\boldsymbol{i}}}^{{\ast\ast}}=\mathrm{diag}\left({\widehat{w}}_{1}^{i},{\widehat{w}}_{2}^{i}\right){\widehat{{\boldsymbol{X}}}}_{{\boldsymbol{i}}}^{{\ast}}\stackrel{P}{\to }diag\left({w}_{1}^{i},{w}_{2}^{i}\right){{\boldsymbol{X}}}_{{\boldsymbol{i}}}^{{\ast}}={{\boldsymbol{X}}}_{{\boldsymbol{i}}}^{{\ast\ast}}\text{ as }{\sum }_{j=1}^{{p}_{i}}I({{\boldsymbol{T}}}_{{\boldsymbol{j}}}^{{\boldsymbol{i}}} \in {\mathbb{T}}_{k})\to \infty$$

for $k=1,2.$ Finally, by an application of the continuous mapping theorem to Eq. (6), we have (${\widehat{\boldsymbol{\alpha }}}_{{\boldsymbol{C}}}^{{\ast}},{\widehat{{\boldsymbol{\beta}}}}_{{\boldsymbol{C}}}^{{\ast}})\stackrel{D}{\to }({\widehat{\boldsymbol{\alpha }}}_{{\boldsymbol{F}}}^{{\ast}},{\widehat{{\boldsymbol{\beta}}}}_{{\boldsymbol{F}}}^{{\ast}})$ as ${\sum }_{j=1}^{{p}_{i}}I({{\boldsymbol{T}}}_{{\boldsymbol{j}}}^{{\boldsymbol{i}}} \in {\mathbb{T}}_{k})\to \infty$ for $k={1,2}$ and $i=1,\ldots ,n.$ CLUSSO is therefore a novel scalar-on-matrix regression technique which regresses a scalar outcome ${y}_{i}$ on the matrix ${\widehat{{\boldsymbol{X}}}}_{{\boldsymbol{i}}}^{{\ast\ast}}$ through the structured lasso. This method allows for unbalanced image feature matrices by averaging features within clusters to estimate image feature-level regression coefficients that are asymptotically error bounded.

In practice, clustering algorithms may or may not be able to identify the perfect clustering solution. Since different clustering algorithms can yield different tubular subgroups and we do not have ground truth tubular subgroup labels, we cannot know which set of clustering tubular subgroup labels is more correct. The candidate clustering algorithm(s) should therefore be chosen based on assumptions about the underlying distribution of the tubular features and/or shape of the underlying subgroups. Several clustering algorithms can also be applied and compared by assessing predictive performance. In Sect. 4, we demonstrate the performance of CLUSSO under several types of misclassification, including different amounts of tubular subgroup misclassification, incorrect specification of the true number of tubular subgroups, and impacts of different choices of the unsupervised clustering algorithm.

## Simulation Study

### Data Generation

For each subject $i=1,\ldots ,n\in \left\{{300,500,700,900,1100,1500,2000}\right\},$ we defined a feature matrix ${{\boldsymbol{X}}}_{{\boldsymbol{i}}}^{{\ast}}$ with two rows and $q\in \{{10,50,100,150,200}\}$ columns ${{\boldsymbol{X}}}_{{\boldsymbol{i}}}^{{\ast}}={\left({{\boldsymbol{X}}}_{{\boldsymbol{i}},\mathbf{1}}^{{\ast}}, {{\boldsymbol{X}}}_{{\boldsymbol{i}},\mathbf{2}}^{{\ast}}\right)}^{T}{\in {\mathbb{R}}}^{2\times q},$ where each row ${{\boldsymbol{X}}}_{{\boldsymbol{i}},{\boldsymbol{k}}}^{{\ast}}$ for $k={1,2}$ of ${{\boldsymbol{X}}}_{{\boldsymbol{i}}}^{{\ast}}$ was a representative tubule for each of the two latent subgroups, and each column was a feature. ${{\boldsymbol{X}}}_{{\boldsymbol{i}},{\boldsymbol{k}}}^{{\ast}}$ represents the population-level tubular feature averages for cluster $k$ and subject $i.$ Elements of ${{\boldsymbol{X}}}_{{\boldsymbol{i}},\mathbf{1}}^{{\ast}}$ were i.i.d drawn from $N({2,1}),$ and elements of ${{\boldsymbol{X}}}_{{\boldsymbol{i}},\mathbf{2}}^{{\ast}}$ were i.i.d drawn from $N({5,3}).$ For each subject, we defined weights ${w}_{k}^{i}$ for $k\in \{{1,2}\}$ that were the relative contributions of each of the two representative tubules for predicting the outcome ${y}_{i}.$ The weight ${w}_{1}^{i}$ was drawn randomly from $\{{0.2,0.3},\ldots ,{0.7,0.8}\}$ for each subject, and ${w}_{2}^{i}=1-{w}_{1}^{i}.$

We calculated scalar outcomes for each subject *i* by ${y}_{i}={\left({\boldsymbol{\alpha }}^{{\ast}}\right)}^{T}{{\boldsymbol{X}}}_{{\boldsymbol{i}}}^{{\ast\ast}}{{\boldsymbol{\beta}}}^{{\ast}}+{\varepsilon }_{i},$ where ${{\boldsymbol{X}}}_{{\boldsymbol{i}}}^{{\ast\ast}}=diag\left({w}_{1}^{i},{w}_{2}^{i}\right){{\boldsymbol{X}}}_{{\boldsymbol{i}}}^{{\ast}}$ and ${\varepsilon }_{i}\sim N(0, {\sigma }^{2}=1).$ Each element of ${\boldsymbol{\alpha }}^{{\ast}}$ and ${{\boldsymbol{\beta}}}^{{\ast}}$ was initially drawn from$\{{1,2},{3,4}\},$ and then we invoked sparsity by randomly sampling ${s}_{{\beta }^{\ast}}\in \{{0.4,0.8}\}$ indices of ${{\boldsymbol{\beta}}}^{{\ast}}$ to be zero. We used the same ${\boldsymbol{\alpha }}^{{\ast}}$ and ${{\boldsymbol{\beta}}}^{{\ast}}$ (with ${s}_{{\beta }^{\ast}}$ indices zeroed out) across simulation settings and simulation repetitions within a given simulation setting. Note that ${{\boldsymbol{X}}}_{{\boldsymbol{i}}}^{{\ast\ast}}\in {\mathbb{R}}^{2\times q}$ is not observed in real practice, so next we generated the observed data.

To determine the number of tubules ${p}_{i}$ observed for each subject, let $M=\left\{{p}_{i}:1\le i\le n\right\},$ with ${\sigma }_{M}^{2}=Var\left(M\right)=5$ and $\mu_M =E\left(M\right)=40.$ Each ${p}_{i}$ was sampled from a discrete uniform distribution $U(a,b)$ of $H$ integers, where $H$ is the largest odd integer less than or equal to $\sqrt{12{\sigma }_{M}^{2}+1},$ which allows us to maintain the desired level of variance of $M.$ To sample the correct mean number tubules on average, we defined *a* and *b* as $a=\mu_M -\frac{H-1}{2}, b=\mu_M +\frac{H-1}{2}.$

Then, the observed ${{\boldsymbol{X}}}_{{\boldsymbol{i}}}={{({\boldsymbol{T}}}_{1}^{{\boldsymbol{i}}}\ldots ,{{\boldsymbol{T}}}_{{{\boldsymbol{p}}}_{{\boldsymbol{i}}}}^{{\boldsymbol{i}}})}^{T}\in {\mathbb{R}}^{{p}_{i}\times q}$ feature matrices were generated by resampling ${p}_{i}$ rows of ${{\boldsymbol{X}}}_{{\boldsymbol{i}}}^{{\ast}}$ with replacement and with Gaussian additive measurement error. Specifically, for each row $j=1,\ldots ,{p}_{i}$ of ${{\boldsymbol{X}}}_{{\boldsymbol{i}}},$ we randomly selected either ${{\boldsymbol{X}}}_{{\boldsymbol{i}},\mathbf{1}}^{{\ast}}$ or ${{\boldsymbol{X}}}_{{\boldsymbol{i}},\mathbf{2}}^{{\ast}}$ with respective probabilities ${w}_{1}^{i}$ and ${w}_{2}^{i}$ of being selected. Then, we used this randomly sampled row ${{\boldsymbol{X}}}_{{\boldsymbol{i}},{\boldsymbol{k}}}^{{\ast}}$ to calculate the *j*th row of ${{\boldsymbol{X}}}_{{\boldsymbol{i}}}$ as ${{\boldsymbol{T}}}_{{\boldsymbol{j}}}^{{\boldsymbol{i}}}={{\boldsymbol{X}}}_{{\boldsymbol{i}},{\boldsymbol{k}}}^{{\ast}} +{{\boldsymbol{\epsilon}}}_{{\boldsymbol{i}},{\boldsymbol{j}}},$ where elements of ${{\boldsymbol{\epsilon}}}_{{\boldsymbol{i}},{\boldsymbol{j}}}\in\mathbb{R}^{1\times q}$ were drawn from $N(0,{\sigma }_{R}^{2}=1).$

To assess the impact of imperfect classification of tubules into subgroups, we conducted additional simulations with ${\sigma }_{R}^{2}\in \{1,6,11,\ldots ,41,46\}$ to imitate feature data with large amounts of noise and therefore increased overlap across the two diseased/non-diseased tubular subgroups. Although we simulated our tubules as independent, tubules may be correlated with each other in real-world histopathology data. Therefore, we also considered additional settings where the tubules were generated in a correlated fashion. We generated tubules from a matrix normal distribution, $\boldsymbol{{X}_{i}^{\ast}}\sim MVN\left(\boldsymbol{\mu} ,\boldsymbol{{\Sigma }_{r}},\boldsymbol{{\Sigma }_{c}}\right)$ [3, 8], where

9 $$\boldsymbol{\mu} =\left[\begin{array}{l}2 \quad \ldots \quad 2\\ 5 \quad \ldots \quad 5\end{array}\right]\in {\mathbb{R}}^{2\times q}$$

$$\begin{array}{c}\#\left(9\right)\end{array}$$

is the mean matrix and

10 $${\boldsymbol{\Sigma }_{r}}=\left[\begin{array}{ll}1& \frac{\sqrt{3}}{2}\\ \frac{\sqrt{3}}{2}& 3\end{array}\right]\in {\mathbb{R}}^{2\times 2}$$

is the row covariance structure, and $\boldsymbol{{\Sigma }_{C}}=I\in {\mathbb{R}}^{q\times q}$ is the identity matrix for the column covariance structure. This tubular image feature generation structure allows tubules from each of the two clusters to have different means, but the choice of $\boldsymbol{{\Sigma }_{r}}$ makes tubules correlated with each other at correlation of 0.5. Additionally, the column covariance structure of $\boldsymbol{{\Sigma }_{C}}$ makes the tubular image features or columns of $\boldsymbol{{X}_{i}^{\ast}}$ independent.

Additionally, we considered misspecification of the number of clusters by generating tubules from three latent subgroups, but having CLUSSO classify tubules into one of two different clusters. Feature values were drawn from $N({2,1}),$ $N\left({5,3}\right),$ and $N({7,3}),$ respectively, for the three subgroups. We set the weight ${w}_{1}^{i}$ to be drawn randomly from $\{{0.2,0.3},\ldots ,0.6\},$ ${w}_{2}^{i}$ to be drawn randomly from $\{0.2,\ldots ,0.8-{w}_{1}^{i}\},$ and then ${w}_{3}^{i}=1-({w}_{1}^{i}+{w}_{2}^{i}).$ To generate subjects’ observed feature matrices ${{\boldsymbol{X}}}_{{\boldsymbol{i}}},$ as with previous simulations, tubules were resampled with measurement error ${\sigma }_{R}^{2}$ from ${{\boldsymbol{X}}}_{{\boldsymbol{i}}}^{{\ast\ast}}$ with resampling probabilities ${w}_{1}^{i},$ ${w}_{2}^{i},$ and ${w}_{3}^{i}$ for the three latent tubular clusters.

We also generated tubules from one latent subgroup, where the latent tubular feature matrices were vectors (${{\boldsymbol{X}}}_{{\boldsymbol{i}}}^{{\ast\ast}}\in {\mathbb{R}}^{1\times q}$), where each value in this single row of feature values was drawn from $N({2,3}).$ However, the clustering algorithms were unable to separate tubules into two clusters, resulting in most tubules being classified into a single cluster, and therefore many subjects being dropped due to not having at least one tubule in each of two clusters.

We used GMM-based clustering based on an expectation maximization (EM) algorithm [20] as the clustering algorithm that was used to generate the CLUSSO results above. We also conducted a set of simulations that compared the use of GMM-based clustering to K-means clustering with CLUSSO to demonstrate the use of different clustering algorithms producing different clustering solutions.

### Alternative Regression Models and Performance Metrics

We compared the performance of CLUSSO to the naïve approach of averaging every feature across the tubule dimension. The naïve averaging procedure produces a feature vector ${\overline{{\boldsymbol{x}}} }_{{\boldsymbol{i}}}=\frac{{\sum }_{j=1}^{{p}_{i}}{{\boldsymbol{T}}}_{{\boldsymbol{j}}}^{{\boldsymbol{i}}}}{{p}_{i}}\in {\mathbb{R}}^{q\times 1}$ for each subject, upon which we can run the regular lasso [22]. We also compared CLUSSO and the naïve approach to the Full Information Structured Lasso, in which we regressed using the structured lasso on the matrices $\left\{{{\boldsymbol{X}}}_{{\boldsymbol{i}}}^{{\ast\ast}}\in {\mathbb{R}}^{2\times q}:i=1,\ldots ,n\right\}$ (or ${{\boldsymbol{X}}}_{{\boldsymbol{i}}}^{{\ast\ast}}\in {\mathbb{R}}^{1\times q}$ for the one latent subgroup and ${{\boldsymbol{X}}}_{{\boldsymbol{i}}}^{{\ast\ast}}\in {\mathbb{R}}^{3\times q}$ for the three latent subgroups simulations). Note that for real datasets, ${{\boldsymbol{X}}}_{{\boldsymbol{i}}}^{{\ast\ast}}$ is not observed. For CLUSSO and the Full Information Structured Lasso, the regularization parameter ${\lambda }_{n}$ was chosen using five-fold cross-validation on the training data to minimize the mean squared error (MSE) over the grid of candidate values {0.5,1,1.5,…,4.5,5}. For each ${\widehat{{\boldsymbol{\beta}}}}^{{\ast}}$ for all three methods, any element of ${\widehat{{\boldsymbol{\beta}}}}^{{\ast}}$ with magnitude smaller than a threshold of 0.001 after L1-normalization of ${\widehat{{\boldsymbol{\beta}}}}^{{\ast}}$ was set to zero.

To compare the performance of the three methods, we considered measures of the true positive rate (TPR) of ${\widehat{{\boldsymbol{\beta}}}}^{{\ast}},$ false positive rate (FPR) of ${\widehat{{\boldsymbol{\beta}}}}^{{\ast}},$ the bias of ${\widehat{{\boldsymbol{\beta}}}}^{{\ast}},$ and the MSE of predicting the outcomes. To measure the TPR of ${\widehat{{\boldsymbol{\beta}}}}^{{\ast}},$ we computed the proportion of nonzero ${{\boldsymbol{\beta}}}^{{\ast}}$ entries which were also identified as nonzero by ${\widehat{{\boldsymbol{\beta}}}}^{{\ast}}.$ For the FPR, we calculated the proportion of zero-valued ${{\boldsymbol{\beta}}}^{{\ast}}$ entries which were incorrectly identified as nonzero by ${\widehat{{\boldsymbol{\beta}}}}^{{\ast}}.$ We computed ${\Vert {\widehat{{\boldsymbol{\beta}}}}^{{\ast}}-{{\boldsymbol{\beta}}}^{{\ast}}\Vert }_{1}$ to estimate the bias of ${\widehat{{\boldsymbol{\beta}}}}^{{\ast}}$ after L1-normalizing both ${\widehat{{\boldsymbol{\beta}}}}^{{\ast}}$ and ${{\boldsymbol{\beta}}}^{{\ast}}.$ For CLUSSO, we also calculated an estimate of the accuracy of the clustering as described in Appendix B of the Supplementary Materials.

We conducted 1000 repetitions per simulation setting, and report the median across simulation repetitions for all performance metrics except the clustering accuracy for which we first calculated the mean clustering accuracy across subjects for a simulation repetition, and then calculated the mean of the means across repetitions. We used means for the clustering accuracy as the clustering accuracy was very similar across simulation repetitions, whereas we used medians for all other performance metrics as their distributions were not symmetric across repetitions.

### Simulation Results

**Fig. 1:**
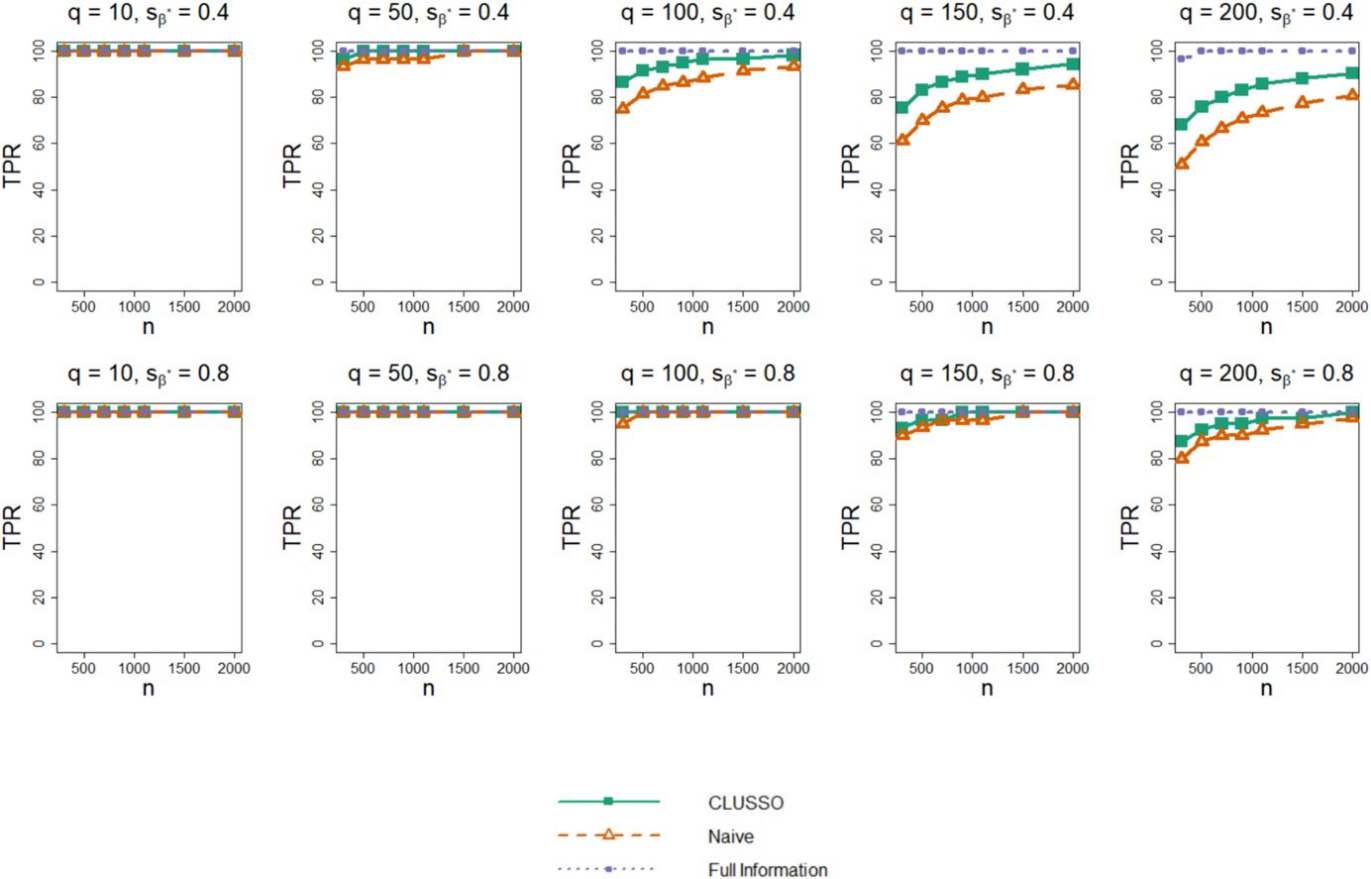
True positive rate of ${\widehat{{\boldsymbol{\beta}}}}^{{\ast}}$ from simulation study. Simulation study results for applying the CLUSSO, naïve, and Full Information Structured Lasso methods, where ${q}$ is the number of features, ${{s}}_{\beta }^{\ast}$ is the proportion of ${{\boldsymbol{\upbeta}}}^{\mathbf{\ast}}$ coefficients which are zero, ${n}$ is the sample size, and the True Positive Rate (TPR) is the proportion of nonzero ${{\boldsymbol{\upbeta}}}^{\mathbf{\ast}}$ entries which were also identified as nonzero by ${\widehat{{\boldsymbol{\upbeta}}}}^{\mathbf{\ast}}.$ All results are averaged by taking the median across the 1000 simulation repetitions

Figure 1 shows the TPR for CLUSSO, naïve, and Full Information Structured Lasso methods. All methods have a non-decreasing TPR as a function of sample size. In the higher sparsity setting ${s}_{{\beta }^{\ast}}=0.8,$ the TPRs of all methods are higher than in the lower sparsity setting ${s}_{{\beta }^{\ast}}=0.4.$ The Full Information Structured Lasso consistently has the highest TPR and CLUSSO has a similar or higher TPR than the naïve method across all simulation settings.

**Fig. 2:**
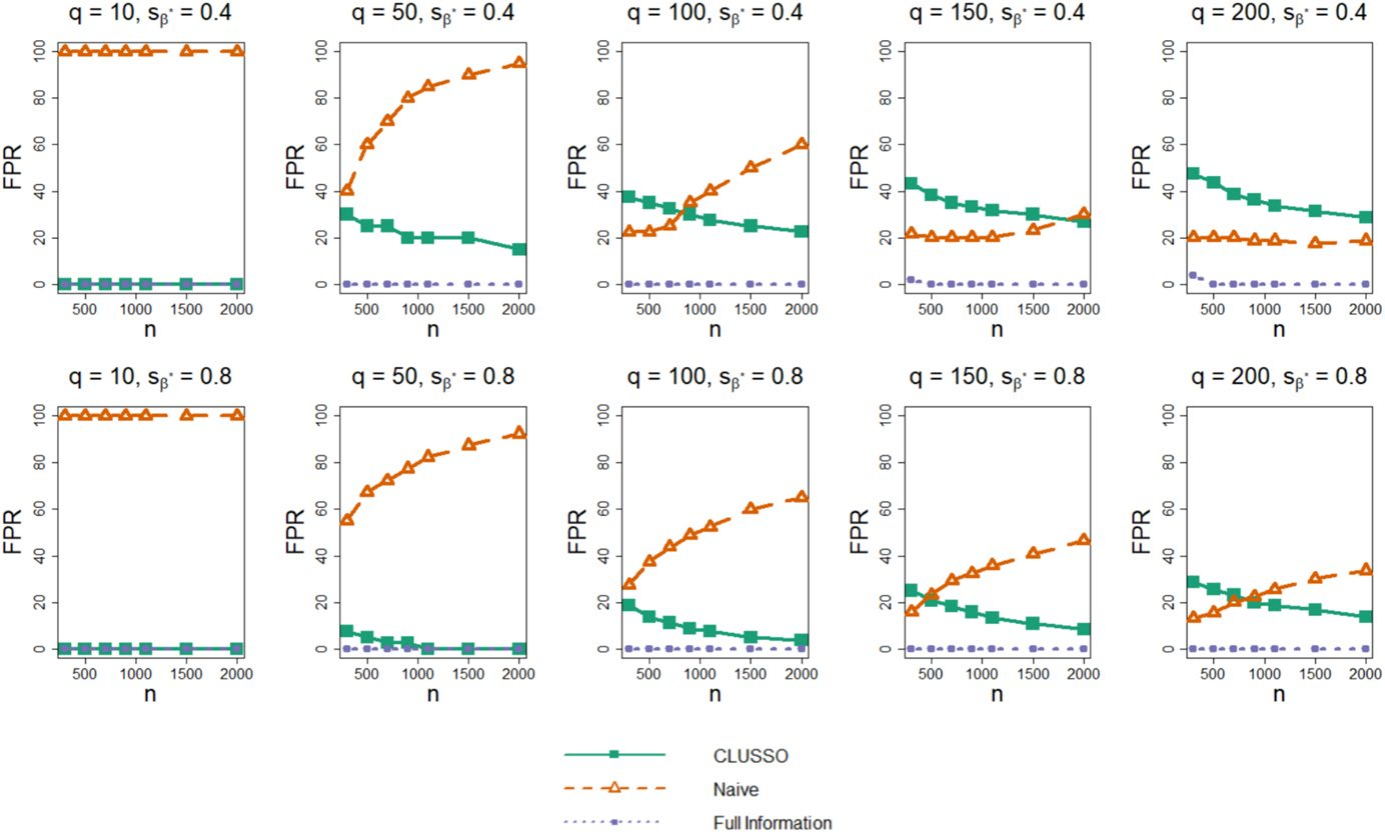
False positive rate of ${\widehat{{\boldsymbol{\beta}}}}^{{\ast}}$ from simulation study. Simulation study results for applying the CLUSSO, naïve, and Full Information Structured Lasso methods, where ${q}$ is the number of features, ${{s}}_{\beta }^{\ast}$ is the proportion of ${{\boldsymbol{\upbeta}}}^{\mathbf{\ast}}$ coefficients which are zero, ${n}$ is the sample size, and the False Positive Rate (FPR) is the proportion of zero-valued ${\upbeta }^{\ast}$ entries which were incorrectly identified as nonzero by ${\widehat{{\boldsymbol{\upbeta}}}}^{\mathbf{\ast}}.$ All results are averaged by taking the median across the 1000 simulation repetitions

Figure 2 shows the FPR across simulation settings. We first observe that the Full Information Structured Lasso correctly recovers the truly important image features with few or no false positive features across all simulation settings. The naïve method has a monotonically non-decreasing FPR with increasing sample size. This pattern is likely due to the naïve lasso fit converging to the ordinary least squares solution as the regularization parameter decreases and more features are selected. In contrast, CLUSSO generally exhibits lower FPRs with increasing sample sizes. As the number of features $q$ increases, the FPR of the naïve method decreases, while the FPR of CLUSSO increases. For the higher sparsity setting ${s}_{{\beta }^{\ast}}=0.8,$ CLUSSO requires a smaller sample size to achieve a lower FPR than the naïve method for the same number of image features.

**Fig. 3:**
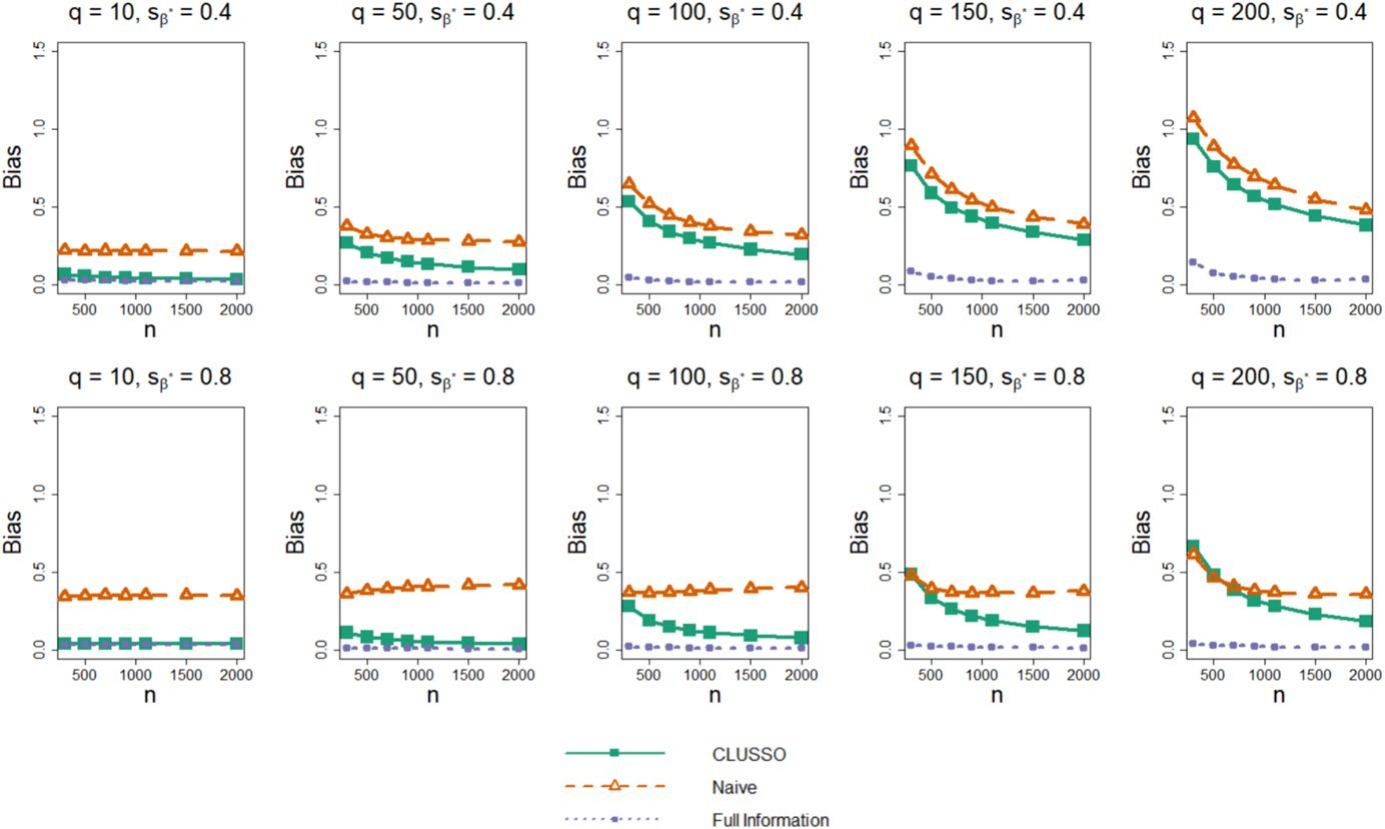
Bias of ${\widehat{{\boldsymbol{\beta}}}}^{{\ast}}$ from simulation study. Simulation study results for applying the CLUSSO, naïve, and Full Information Structured Lasso methods, where ${q}$ is the number of features, ${{s}}_{\upbeta }^{\ast}$ is the proportion of ${{\boldsymbol{\upbeta}}}^{\mathbf{\ast}}$ coefficients which are zero, ${n}$ is the sample size, and the bias is calculated as ${\Vert {\widehat{{\boldsymbol{\upbeta}}}}^{\mathbf{\ast}}-{{\boldsymbol{\upbeta}}}^{\mathbf{\ast}}\Vert }_{1}$ after L1-normalizing both ${\widehat{{\boldsymbol{\upbeta}}}}^{\mathbf{\ast}}$ and ${{\boldsymbol{\upbeta}}}^{\mathbf{\ast}}.$ All results are averaged by taking the median across the 1000 simulation repetitions

**Fig. 4:**
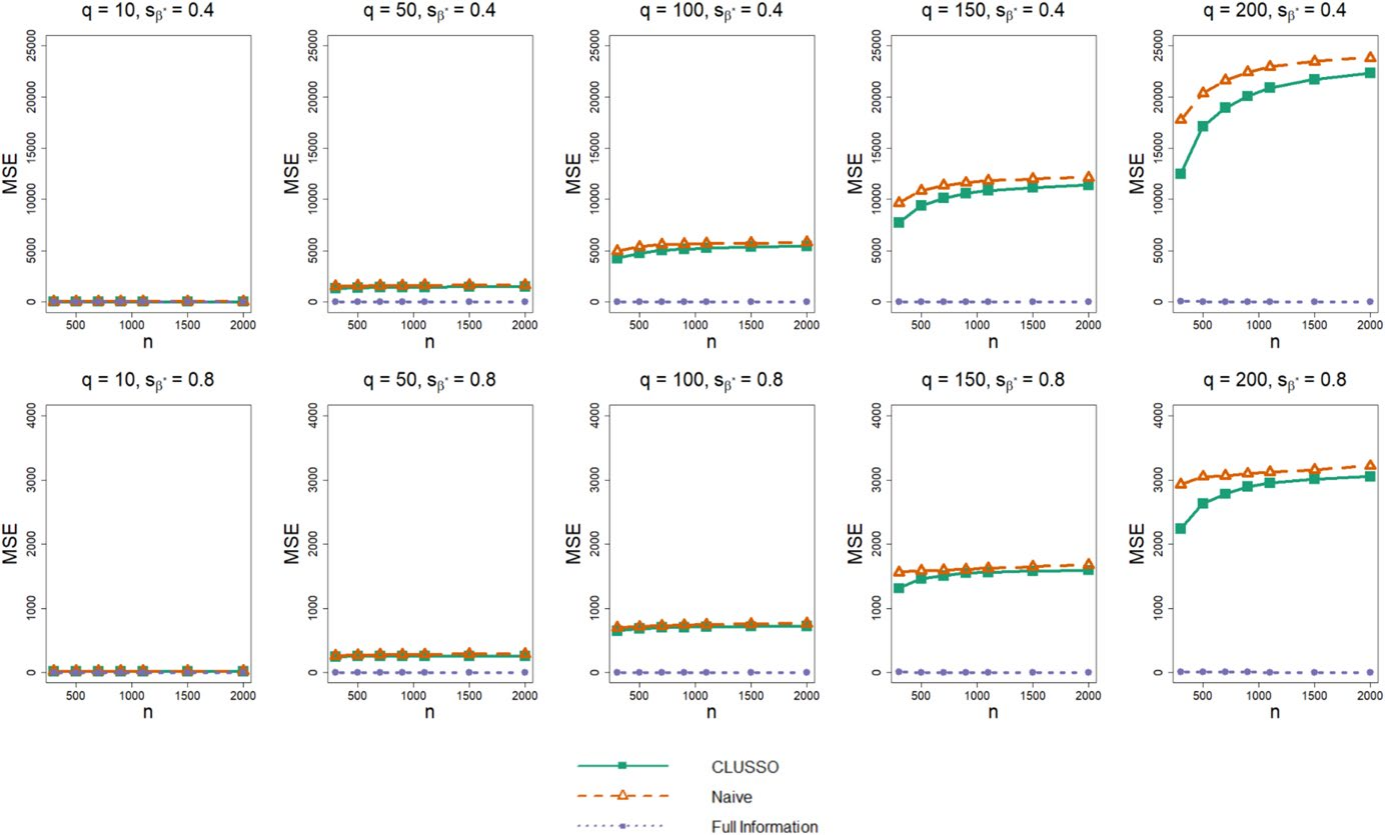
Prediction mean squared error from simulation study. Simulation study results for applying the CLUSSO, naïve, and Full Information Structured Lasso methods, where ${q}$ is the number of features, ${{s}}_{\upbeta }^{\ast}$ is the proportion of ${{\boldsymbol{\upbeta}}}^{\mathbf{\ast}}$ coefficients which are zero, ${n}$ is the sample size, and the mean squared error is denoted by MSE. All results are averaged by taking the median across the 1000 simulation repetitions

In terms of the bias of ${\widehat{{\boldsymbol{\beta}}}}^{{\ast}},$ we find that CLUSSO often has a lower bias than the naïve approach, and the biases of both methods are larger than those of the Full Information Structured Lasso across all simulation settings (Fig. 3). We also observe in Fig. 4 that CLUSSO has a comparable MSE to the naïve method. However, both methods have a higher MSE than the Full Information Structured Lasso across all simulation settings.

**Fig. 5:**
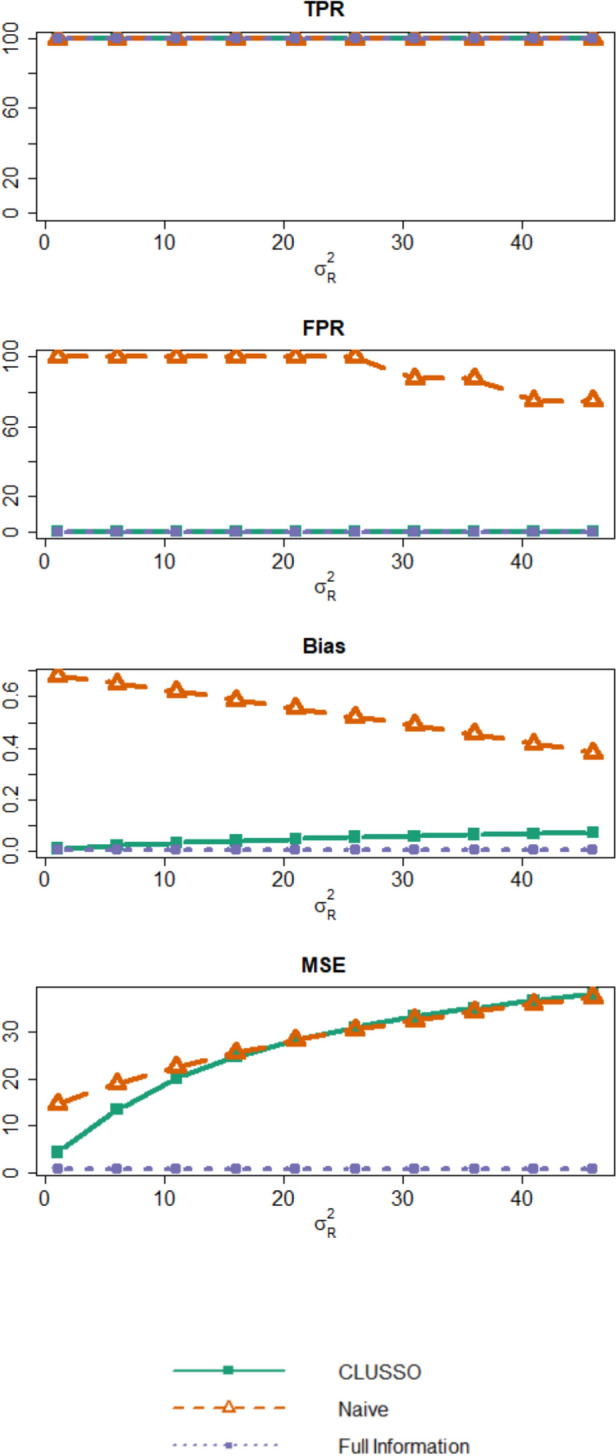
Simulation study results from varying resampling variability ${\sigma }_{R}^{2}.$ Simulation study results for applying the CLUSSO, naïve, and Full Information Structured Lasso methods. For these simulations where ${q}=10$ is the number of features, ${{s}}_{\upbeta }^{\ast}=0.8$ is the proportion of ${{\boldsymbol{\upbeta}}}^{\mathbf{\ast}}$ coefficients which are zero, ${n}=500$ is the sample size, and ${\upsigma }_{{R}}^{2}\in \{{1,6},{11,16,21,26,31,36,41,46}\}$ is the resampling variability. All results are averaged by taking the median across the 1000 simulation repetitions

Figure 5, Supplementary Materials Tables 1–2, and Supplementary Figs. 1–4 show simulation results from changing the resampling variability ${\sigma }_{R}^{2}.$ We first note that under small levels of measurement error in the observed tubular features, GMM-based clustering achieves near perfect clustering accuracy (Supplementary Materials Table 1). Supplementary Materials Table 1 also illustrates that the clustering accuracy of GMM-based clustering steadily decreases from about 100% to around 74% as ${\sigma }_{R}^{2}$ increases from 1 to 46. From Fig. 5, we see that while all methods have a similar TPR at close to 100%, the naïve method had an inflated FPR relative to CLUSSO and the Full Information Structured Lasso for increasing ${\sigma }_{R}^{2}.$ All methods exhibited similar bias values across all simulation settings with increasing ${\sigma }_{R}^{2}.$ For the MSE, the naïve method and CLUSSO were nearly identical across all simulation settings but larger than the Full Information Structured Lasso across all simulation settings with increasing ${\sigma }_{R}^{2}.$ From these simulation results, we see that even when the clustering into latent subgroups is inaccurate, using CLUSSO is better than not using clustering with the naive approach for recovering the important features. Additionally, when the tubules are correlated in these simulation settings (Supplementary Fig. 1), we see that CLUSSO still has comparable TPRs and MSEs with lower FPRs and biases relative to the naïve approach. Therefore, CLUSSO was not sensitive to correlations between tubules.

**Table 1:** Estimated coefficient magnitudes of selected tubular image features for predicting kidney function

Feature	CLUSSO ${\widehat{{\boldsymbol{\beta}}}}^{{\ast}}$ coefficient magnitudes	Naïve method ${\widehat{{\boldsymbol{\beta}}}}^{{\ast}}$ coefficient magnitudes
Tubular epithelium + lumen area	1	0
Lumen average thickness	0	0.009
Tubular basement membrane smoothness	0	0.838
Tubular basement membrane diameter-to-tubule diameter ratio	0	0.153

L1-normalized regression coefficients for CLUSSO and the naïve approach when trained on the tubular digital image features in the NEPTUNE data analyzed in Sect. 5

Supplementary Materials Fig. 2 illustrates that CLUSSO has similar TPRs, biases, and MSEs, but consistently lower FPRs relative to the naïve approach when there are three latent tubular clusters instead of two. Therefore, CLUSSO demonstrates some robustness to misspecification of the true number of latent tubular clusters. We do not provide comparable results for when there is only one latent tubular cluster due to the large proportions of tubules that get classified into one cluster for each simulation repetition (Supplementary Materials Table 2). In practice, such clustering results may provide evidence that tubules are not drawn from a two-component mixture distribution and the naïve approach may be sufficient in these situations.

For K-means clustering, Supplementary Materials Table 1 shows that this clustering algorithm achieves similar levels of clustering accuracy as GMM-based clustering across different values of ${\sigma }_{R}^{2}.$ However, the confusion matrix that compares clustering labels of the GMM-based clustering and the K-means clustering for a simulation repetition under high resampling variability (Supplementary Materials Fig. 3) indicates imperfect concordance (86.8%) of the clustering labels between algorithms. Thus, there is potential for similar levels of clustering accuracy to be achieved with different unsupervised clustering methods which do not produce identical clustering labels for the tubules. Yet, Supplementary Materials Fig. 4 demonstrates that CLUSSO can achieve similar TPRs and MSEs with lower FPRs and biases to the naïve approach for increasing ${\sigma }_{R}^{2}.$ These simulation results show that CLUSSO is not sensitive to the choice of clustering algorithms.

## Real Data Analysis: Digital Pathology Biomarkers for Glomerular Disease

NEPTUNE and CureGN are multisite observational cohort studies of subjects with glomerular disease, enrolled at the time of their first clinically indicated renal biopsy for NEPTUNE [6] or up to five years after the first diagnostic biopsy [16]. Subject demographics and serum creatinine were utilized to calculate the estimated glomerular filtration rate (eGFR), a measure of kidney function, at the time of biopsy using the CKD-EPI formula [11] for adults over 25 and the U25 formula for children and adults under 25 [19]. One WSI of Periodic acid-Schiff (PAS)-stained renal biopsy tissue was obtained from each of 250 NEPTUNE study subjects and 261 CureGN subjects. A previously trained deep learning model was applied for the automatic segmentation of tubules. After segmentation of each tubule from each WSI, fifty-seven tubular image features were computed as listed in Supplementary Materials Table 3. These features are hand-crafted size and shape characteristics (area, diameter, thickness, smoothness, average distance, and ratios of these values) that were calculated on different parts of the tubule including the nucleus, lumen, tubular epithelium, and tubular basement membrane. Several of these features were also computed using the entire tubule (e.g., tubule area). The median number of tubules per subject in the NEPTUNE and CureGN cohorts were 929 and 900 with interquartile ranges of (570, 1377) and (583, 1404), respectively (Supplementary Materials Fig. 5). The primary aim of our analysis was to identify tubular image features that were predictive of eGFR at biopsy.

We set the number of tubular clusters to $G=2$ for separating tubules into subgroups, and searched over the grid of $\{{0.1,0.2},\ldots ,{19.9,20}\}$ to choose the regularization parameter using five-fold cross-validation with MSE as the minimization criterion for the CLUSSO model trained on the NEPTUNE cohort. We found that CLUSSO chose the regularization parameter $\lambda =19.7,$ which provides confidence that the range of regularization parameters was large enough. Additionally, we picked the ${\widehat{{\boldsymbol{\beta}}}}_{{\boldsymbol{C}}}^{{\ast}}$ for CLUSSO that minimized the CLUSSO objective function for five different random choices of initializations for ${\boldsymbol{\alpha }}^{{\ast}}$ and ${{\boldsymbol{\beta}}}^{{\ast}},$ as the algorithm to find the solution of the structured lasso [27] converges to a stationary point rather than the global minimizer, and the authors advised using several random initializations of ${\boldsymbol{\alpha }}^{{\ast}}$ and ${{\boldsymbol{\beta}}}^{{\ast}}$ if converging to a global minimizer is an issue. For the naïve approach, the regularization parameter ${\lambda }_{\rm naive}=1.92$ for the NEPTUNE cohort was chosen using five-fold cross-validation to minimize the MSE over a grid of 100 values with maximum ${\lambda }_{\max}=17.47,$ the smallest $\lambda$ such that all estimated coefficients are zero, and minimum ${\lambda }_{\rm min}={\lambda }_{\rm max} \times {10}^{-4},$ as proposed in Friedman et al. [5].

Table 1 shows the estimated magnitudes of the regression coefficients for image feature effects that were nonzero for either CLUSSO or the naïve approach when trained on the NEPTUNE cohort. All other features provided in Supplementary Materials Table 3 that are not listed in Table 1 had estimated regression coefficient magnitudes of zero for both CLUSSO and the naïve approach when trained on the NEPTUNE data. We did not report the signs of the estimated regression coefficients as the product of ${\boldsymbol{\alpha }}^{{\ast}}$ and ${{\boldsymbol{\beta}}}^{{\ast}}$ is involved when optimizing the objective function for CLUSSO, so their signs are not identifiable. CLUSSO only selected tubular epithelium and lumen area as being important for the prediction of eGFR at time of biopsy. In contrast to CLUSSO, the naïve approach selected lumen average thickness, tubular basement membrane smoothness, as well as tubular basement membrane diameter-to-tubule diameter ratio.

To compare prediction performance of the CLUSSO model and naïve approach, we computed MSEs. When applying the estimated regression coefficients from both models to predict eGFR on the same cohort of NEPTUNE subjects for which the models were trained, the naïve approach produced an MSE of 779 while CLUSSO produced an MSE of 757. The reduction in MSE (3%) provided by CLUSSO relative to the naïve approach indicates that CLUSSO provides a slight improvement in predicting eGFR among subjects in the NEPTUNE cohort.

For external validation of these selected image features and prediction models, we applied the fitted naïve approach and CLUSSO models to predict eGFR for the CureGN subjects. An important consideration for applying CLUSSO to an independent test set is that it is unclear which ordering of tubular clusters in the test set feature matrices best correspond to the tubular clusters in the training set used to estimate cluster-level effects ${\widehat{\boldsymbol{\alpha }}}_{{\boldsymbol{C}}}^{{\ast}}.$ For instance, suppose the first component of ${\widehat{\boldsymbol{\alpha }}}_{{\boldsymbol{C}}}^{{\ast}}$ reflects the cluster-level effect of tubules labeled as “1” by an unsupervised clustering algorithm in the training set, and the second component of ${\widehat{\boldsymbol{\alpha }}}_{{\boldsymbol{C}}}^{{\ast}}$ reflects the cluster-level effect of tubules labeled as “2.” Applying this same unsupervised clustering algorithm to the testing data will also produce tubule labels of “1” or “2,” but test set cluster “1” may actually have more similar image features to training set cluster “2.”

Therefore, both possible correspondences of tubular clustering labels between the training and testing data should be considered when applying the CLUSSO model to independent testing data. For the CureGN cohort, the naïve approach yielded an MSE of 1177, while CLUSSO yielded MSEs of 1094 or 6205 for the two different correspondences of tubular clustering labels between the training and testing data. The smaller MSE of 1094 for CLUSSO more likely reflects clusters of tubules for the testing data that are more similar to those of the training data, and in general the correspondence of clusters between the training and testing data that yields the smallest MSE in the testing data should be used to obtain the desired predictions. Similarly, if different MSEs are obtained using CLUSSO from different choices of clustering algorithms, then the tubular clustering labels which yield the smallest MSE with CLUSSO should be taken as the better tubular clustering labels.

The reduction in MSE provided by CLUSSO relative to the naïve approach (7%) when applied to the CureGN cohort indicates that the biomarker generated by CLUSSO is more generalizable to different cohorts of subjects with glomerular disease and has improved utility in predicting kidney function. As the CLUSSO model only needed one image feature to predict kidney function with better predictive accuracy to the naïve approach which used three image feature biomarkers, CLUSSO may allow for a more parsimonious and computationally simple method of predicting kidney function in subjects with glomerular disease. The tubular epithelium + lumen area feature selected by CLUSSO is consistent with histopathological changes associated with tubular atrophy. For example, Lusco et al. [15] described contraction of tubular lumen and flattened tubular epithelial cells when there is tubular atrophy, which would decrease the tubular epithelium and lumen area.

## Discussion

Computer-extracted image features allow for the characterization of tubules from WSIs and could potentially be used as image-based biomarkers for predicting clinical outcomes. However, existing methods are limited for analyzing these data and selecting the features that are most predictive of outcomes. Although deep learning algorithms can be trained to predict outcomes, these algorithms often do not yield active sets of features driving outcome prediction nor feature rankings. Scalar-on-matrix regression models offer a promising alternative for selecting important features but were not previously able to handle unordered and differing numbers of tubules across subjects. Therefore, we developed CLUSSO, a novel scalar-on-matrix regression technique, which provides feature selection and feature rankings in a way that accounts for the matrix-valued data structure of features measured for each of a number of tubules, allowing for differing numbers of tubules per subject.

We demonstrated through a simulation study that CLUSSO can better identify features that are most informative of an outcome and even improve our ability to predict the outcome compared to a naïve averaging strategy. CLUSSO identified the informative features with a greater TPR and lower FPR than the naïve method across almost all simulation settings, particularly for higher sparsity levels ${s}_{{\beta }^{\ast}}$ and smaller numbers of features $q.$ While a greater TPR ensures important features are not missed, the potential impacts of a lower FPR are also substantial, e.g., by decreasing costs or increasing power in subsequent studies aimed at validating the selected features. There were many different simulation settings in which CLUSSO reduced the FPR relative to the naïve approach by at least 0.30, which is comparable to other methods that have been designed to reduce false discovery rates [21]. Additionally, models with fewer selected features due to fewer false discoveries are simpler to interpret and more likely to be generalizable.

CLUSSO also had smaller FPRs than the naïve method across different resampling variabilities ${\sigma }_{R}^{2}$ when GMM-based clustering was used. Therefore, even when the latent tubular clusters were less distinguishable, CLUSSO was still robust in selecting important features. CLUSSO was also robust to correlated tubules, a misspecified number of latent tubular clusters or choice of unsupervised clustering algorithm. The better feature selection performance of CLUSSO compared to the naïve method also did not come at a cost of worse prediction, as CLUSSO had similar or lower MSEs than the naïve method across all simulation settings.

There are numerous potential extensions of CLUSSO to accommodate different types of predictors and outcomes. Rather than using a continuous outcome, a binary outcome could be used in a matrix variate logistic model as defined by Hung and Wang [10], for which an L1 penalty on the column-level effects is used in the objective function. Similarly, an L1-penalized Cox proportional hazards model for matrix-valued predictors could be developed to handle survival outcomes. Further, CLUSSO could be adjusted for predictors for which we observe one scalar per subject by adding these predictors in the sum of squared errors component of the CLUSSO objective function. As the lasso tends to select only one or a few features that are highly correlated and associated with the outcome, alternative L1-penalized regression techniques such as the random lasso [23] could be used in place of the lasso when minimizing the objective function for CLUSSO to better handle correlated features. The work of He et al. [9] could be employed to control the false discovery rate of CLUSSO at a pre-specified threshold.

There may be an opportunity to extend the framework of Ou-Yang et al. [18], which uses a sparse penalty on the coefficient tensor in a tensor regression, to generalize our proposed methodology from two-dimensional unbalanced predictors to unbalanced arrays of more than two dimensions. Another interesting area for future development could be extending CLUSSO to settings where different subjects have different numbers of latent clusters. Finally, as misclassification of tubules results in observed CLUSSO tubular feature matrices with measurement error, investigating the performance of CLUSSO under different measurement error models is an additional direction for future research.

As digital pathology and image feature extraction becomes increasingly common, we propose the use of CLUSSO to analyze these unique data and select a sparse set of image features which are predictive of outcomes. Since CLUSSO can better identify the active set of image features, we have improved ability to discover novel image-based biomarkers that can ultimately enhance diagnosis and prognosis for kidney diseases and other medical conditions with tissue biopsy samples.

## Supplementary Information

Supplementary Materials Appendices, Tables, and Figures referenced in Sects. 2–5 are available with this paper at the Statistics in Biosciences website. Code is provided which walks through applying CLUSSO and the naïve approach to an illustrative and synthetic dataset.

## Supplementary Information

Below is the link to the electronic supplementary material.

Supplementary file1 (DOCX 167 KB)

## Data Availability

The data used in the application of this study were obtained from the Nephrotic Syndrome Study Network (NEPTUNE) and Cure Glomerulonephropathy (CureGN). Data sharing requires ancillary study approval and a data use agreement, which can be requested from the NEPTUNE Data Analysis and Coordinating Center (DACC) and CureGN Data Coordinating Center (DCC), https://www.neptune-study.org/ and https://www.dev-curegn.org/.
