# Supplementary material for: Novel Scalar-on-matrix Regression for Unbalanced Feature Matrices
Source: Stat Biosci. 2025 Mar 5;18(1):192–213. doi: 10.1007/s12561-025-09476-7 (PMC12456458; doi:10.1007/s12561-025-09476-7)
Supplement: Supplementary file 1 — Supplementary file1 (DOCX 167 KB) [file 12561_2025_9476_MOESM1_ESM.docx]

Supplementary Materials to Novel Scalar-on-matrix Regression for Unbalanced Feature Matrices

Statistics in Biosciences

**Jeremy Rubin**

Department of Biostatistics, Epidemiology, and Informatics, University of Pennsylvania Perelman School of Medicine, Philadelphia, PA, USA.

**Fan Fan**

Department of Biomedical Engineering, Emory University and Georgia Institute of Technology**,** Atlanta, GA, USA.

**Laura Barisoni**

Division of AI and Computational Pathology, Department of Pathology, Duke University, Durham, NC, USA.

Division of Nephrology, Department of Medicine, Duke University, Durham, NC, USA.

**Andrew R. Janowczyk**

Oncology and Pathology Departments, Geneva University Hospitals, Geneva, Switzerland.

Department of Biomedical Engineering, Emory University and Georgia Institute of Technology**,** Atlanta, GA, USA.

**Jarcy Zee**

Department of Biostatistics, Epidemiology, and Informatics, University of Pennsylvania Perelman School of Medicine, Philadelphia, PA, USA.

Pediatrics, The Children’s Hospital of Philadelphia Research Institute, Philadelphia, PA, USA. Email: [jarcy.zee@Pennmedicine.upenn.edu](mailto:jarcy.zee@Pennmedicine.upenn.edu)

Address: 210 Blockley Hall, 423 Guardian Drive, Philadelphia, PA 19104

**Supplementary Materials Appendix A**: Illustrative Example for Interpretation of $\boldsymbol{\alpha}^{\boldsymbol{*}}$ and $\boldsymbol{\beta}^{\boldsymbol{*}}$

Suppose that each subject has a feature matrix $\boldsymbol{X}_{\boldsymbol{i}}\in\mathbb{R}^{2\times3}$.

Then, in the absence of an error term, each $y_{i}$ is comprised of the following product:

$$y_{i}=\left[ \alpha_{1}^{*}, \alpha_{2}^{*} \right]\left[ \begin{matrix} X_{11} & X_{12} & X_{13} \\ X_{21} & X_{22} & X_{23} \end{matrix} \right]\left[ \begin{matrix} \beta_{1}^{*} \\ \beta_{2}^{*} \\ \beta_{3}^{*} \end{matrix} \right]$$

$$\begin{aligned} =\alpha_{1}^{*}X_{11}\beta_{1}^{*}+\alpha_{2}^{*}X_{21}\beta_{1}^{*}+\alpha_{1}^{*}X_{12}\beta_{2}^{*}+\alpha_{2}^{*}X_{22}\beta_{2}^{*}+\alpha_{1}^{*}X_{13}\beta_{3}^{*}+\alpha_{2}^{*}X_{23}\beta_{3}^{*}. \#\left( 1 \right) \end{aligned}$$

The effect on $y_{i}$ of a 1-unit increase in $X_{11}$ would be:

$$\alpha_{1}^{*}{(X}_{11}+1)\beta_{1}^{*}+\alpha_{2}^{*}X_{21}\beta_{1}^{*}+\alpha_{1}^{*}X_{12}\beta_{2}^{*}+\alpha_{2}^{*}X_{22}\beta_{2}^{*}+\alpha_{1}^{*}X_{13}\beta_{3}^{*}+\alpha_{2}^{*}X_{23}\beta_{3}^{*}$$

=
$\alpha_{1}^{*}X_{11}\beta_{1}^{*}+\alpha_{1}^{*}\beta_{1}^{*}+\alpha_{2}^{*}X_{21}\beta_{1}^{*}+\alpha_{1}^{*}X_{12}\beta_{2}^{*}+\alpha_{2}^{*}X_{22}\beta_{2}^{*}+\alpha_{1}^{*}X_{13}\beta_{3}^{*}+\alpha_{2}^{*}X_{23}\beta_{3}^{*}$*.*

As the signs of $\alpha_{1}^{*}$ and $\beta_{1}^{*}$ are not identifiable by the structured lasso, at best we can say a change in one-unit of $X_{11}$ results in an $\alpha_{1}^{*}\beta_{1}^{*}$ magnitude change in $y_{i}$. We are unable to say whether the change will be positive or negative because we do not know if $\alpha_{1}^{*}$ and $\beta_{1}^{*}$ have the same sign. Furthermore, normalization of the coefficient vectors during parameter estimation implies that reversing the normalization is necessary to interpret these relative changes as magnitudes of the changes in $y_{i}$. Therefore, **a one-unit change in the cluster-**$\boldsymbol{k}$ **averaged and weighted feature value** $\boldsymbol{j}$ **will result in a relative** $\boldsymbol{\alpha}_{\boldsymbol{k}}^{\boldsymbol{*}}\boldsymbol{\beta}_{\boldsymbol{j}}^{\boldsymbol{*}}$ **change in** $\boldsymbol{y}_{\boldsymbol{i}}$.

Note that by rearranging the terms in (1), we can see that (1) can be written as a weighted sum of inner products of $\boldsymbol{\alpha}^{\boldsymbol{*}}$ with columns of $\boldsymbol{X}_{\boldsymbol{i}}$:

$$\alpha_{1}^{*}X_{11}\beta_{1}^{*}+\alpha_{2}^{*}X_{21}\beta_{1}^{*}+\alpha_{1}^{*}X_{12}\beta_{2}^{*}+\alpha_{2}^{*}X_{22}\beta_{2}^{*}+\alpha_{1}^{*}X_{13}\beta_{3}^{*}+\alpha_{2}^{*}X_{23}\beta_{3}^{*}$$

$$=\left( \alpha_{1}^{*}X_{11}+\alpha_{2}^{*}X_{21} \right)\beta_{1}^{*}+\left( \alpha_{1}^{*}X_{12}+\alpha_{2}^{*}X_{22} \right)\beta_{2}^{*}+\left( \alpha_{1}^{*}X_{13}+\alpha_{2}^{*}X_{23} \right)\beta_{3}^{*}$$

$= <\boldsymbol{X}_{\boldsymbol{,1}},\boldsymbol{\alpha}^{\boldsymbol{*}}>\beta_{1}^{*}+ <\boldsymbol{X}_{\boldsymbol{,2}},\boldsymbol{\alpha}^{\boldsymbol{*}}>\beta_{2}^{*}+ <\boldsymbol{X}_{\boldsymbol{,3}},\boldsymbol{\alpha}^{\boldsymbol{*}}>\beta_{3}^{*}$ (2)

where $<\boldsymbol{a},\boldsymbol{b}>$ denotes the inner product of $\boldsymbol{a}$ and $\boldsymbol{b}$, and $\boldsymbol{X}_{\boldsymbol{,j}}$ denotes column $j\in\{1,2,3\}$ of $\boldsymbol{X}_{\boldsymbol{i}}$.

Now, let’s consider what happens when we increase each value in the first column of $\boldsymbol{X}_{\boldsymbol{i}}$ by 1 using (2):

$$<{(\boldsymbol{X}}_{\boldsymbol{,1}}+\boldsymbol{1}),\boldsymbol{\alpha}^{\boldsymbol{*}}>\beta_{1}^{*}+ <\boldsymbol{X}_{\boldsymbol{,2}},\boldsymbol{\alpha}^{\boldsymbol{*}}>\beta_{2}^{*}+ <\boldsymbol{X}_{\boldsymbol{,3}},\boldsymbol{\alpha}^{\boldsymbol{*}}>\beta_{3}^{*}$$

$$= <\boldsymbol{X}_{\boldsymbol{,1}},\boldsymbol{\alpha}^{\boldsymbol{*}}>\beta_{1}^{*}+\boldsymbol{<1,}\boldsymbol{\alpha}^{\boldsymbol{*}}\boldsymbol{>}\beta_{1}^{*}+ <\boldsymbol{X}_{\boldsymbol{,2}},\boldsymbol{\alpha}^{\boldsymbol{*}}>\beta_{2}^{*}+ <\boldsymbol{X}_{\boldsymbol{,3}},\boldsymbol{\alpha}^{\boldsymbol{*}}>\beta_{3}^{*}$$

$= <\boldsymbol{X}_{\boldsymbol{,1}},\boldsymbol{\alpha}^{\boldsymbol{*}}>\beta_{1}^{*}+\beta_{1}^{*}\Sigma_{k=1}^{2}\alpha_{k}^{*}+ <\boldsymbol{X}_{\boldsymbol{,2}},\boldsymbol{\alpha}^{\boldsymbol{*}}>\beta_{2}^{*}+ <\boldsymbol{X}_{\boldsymbol{,3}},\boldsymbol{\alpha}^{\boldsymbol{*}}>\beta_{3}^{*}$ (3)

Therefore, a one-unit increase in each entry of column 1 of $\boldsymbol{X}_{\boldsymbol{i}}$ will result in a $\beta_{1}^{*}\Sigma_{k=1}^{2}\alpha_{k}^{*}$ relative change in $y_{i}$. More generally, **a one-unit increase for all feature** $\boldsymbol{j}$ **cluster-averages will result in a relative change in** $\boldsymbol{y}_{\boldsymbol{i}}$ **of** $\boldsymbol{\beta}_{\boldsymbol{j}}^{\boldsymbol{*}}\boldsymbol{\Sigma}_{\mathbf{k=1}}^{\mathbf{G}}\boldsymbol{\alpha}_{\boldsymbol{k}}^{\boldsymbol{*}}$. We are unable to say in which direction $y_{i}$ will change as we do not know the sign of $\beta_{j}^{*}$ and interpretation of the magnitude of $\beta_{j}^{*}\Sigma_{k=1}^{G}\alpha_{k}^{*}$would require reversal of the normalizations of both coefficient vectors. However, for one-unit increases for all cluster-averages of different features, we see that their corresponding relative changes in $y_{i}$ will differ only for different values of $\beta_{j}^{*}$. Therefore, the magnitudes of these $\beta_{j}^{*}$ components are the only necessary information to rank the image features by their relative effects on the outcome.

Due to lack of sign identifiability and normalization, the primary goal of the Full Information Structured Lasso is thus to identify the active set of important image features and the rankings of the features in terms of importance, as opposed to making conclusions about the estimated effect sizes of the image feature.

**Supplementary Materials Appendix B**: Clustering accuracy

We know that every object $j=1,\ldots,p_{i}$ in $\boldsymbol{X}_{\boldsymbol{i}}$ has the cluster assignment corresponding to row $r\in\left\{ 1,\ldots,G \right\}$ of $\boldsymbol{X}_{\boldsymbol{i}}^{\boldsymbol{*}}$, $\boldsymbol{X}_{\boldsymbol{i,k}}^{\boldsymbol{*}}$, which was resampled to generate $\boldsymbol{X}_{\boldsymbol{i,j}}$. Denote the true set of tubular cluster labels as $\mathbb{K}$. Then, let $\hat{\mathbb{K}}$ be the estimated set of object cluster labels based on the clustering step of CLUSSO, where each cluster label can take value $A$ or $B$. Since we do not know if estimated cluster $A$ corresponds to true cluster 1 or true cluster 2, we must consider both possibilities.

As we only have two cluster labels, there are only two possible correct correspondences of labels between cluster assignments in $\mathbb{K}$ and those in $\hat{\mathbb{K}}$ for a given object $\boldsymbol{T}_{\boldsymbol{j}}^{\boldsymbol{i}}$ for $j=1,\ldots,p_{i}$:

**Correspondence 1**: $\boldsymbol{T}_{\boldsymbol{j}}^{\boldsymbol{i}}$ belongs to cluster $k=1$ in $\mathbb{K}$ with matching cluster assignment $A$ in $\hat{\mathbb{K}}$ or

$\boldsymbol{T}_{\boldsymbol{j}}^{\boldsymbol{i}}$ belongs to cluster $k=2$ in $\mathbb{K}$ with matching cluster assignment of $B$ in $\hat{\mathbb{K}}$

**Correspondence 2**: $\boldsymbol{T}_{\boldsymbol{j}}^{\boldsymbol{i}}$ belongs to cluster $k=1$ in $\mathbb{K}$ with matching cluster assignment $B$ in $\hat{\mathbb{K}}$ or

$\boldsymbol{T}_{\boldsymbol{j}}^{\boldsymbol{i}}$ belongs to cluster $k=2$ in $\mathbb{K}$ with matching cluster assignment of $A$ in $\hat{\mathbb{K}}$

Let $\mathbb{K(}\boldsymbol{T}_{\boldsymbol{j}}^{\boldsymbol{i}})$ denote the cluster assignment of object $j$ for subject $i$ in $\mathbb{K}$, and let $\hat{\mathbb{K}}(\boldsymbol{T}_{\boldsymbol{j}}^{\boldsymbol{i}})$ symbolize the respective object label in $\hat{\mathbb{K}}$. As the clustering is unsupervised and the correct correspondence of object cluster assignments may change in different simulations, we define the clustering accuracy, $c_{k}$, for subject $i$ as:

$$c_{k}=\max\left\{ \begin{aligned} \frac{\sum_{j=1}^{p_{i}} I\left\{ \left( \mathbb{K}\left( \boldsymbol{T}_{\boldsymbol{j}}^{\boldsymbol{i}} \right)=1 and \hat{\mathbb{K}}\left( \boldsymbol{T}_{\boldsymbol{j}}^{\boldsymbol{i}} \right)=A \right) or \left( \mathbb{K}\left( \boldsymbol{T}_{\boldsymbol{j}}^{\boldsymbol{i}} \right)=2 and \hat{\mathbb{K}}\left( \boldsymbol{T}_{\boldsymbol{j}}^{\boldsymbol{i}} \right)=B \right) \right\}}{p_{i}}, \\ \frac{\sum_{j=1}^{p_{i}} I\left\{ \left( \mathbb{K}\left( \boldsymbol{T}_{\boldsymbol{j}}^{\boldsymbol{i}} \right)=1 and \hat{\mathbb{K}}\left( \boldsymbol{T}_{\boldsymbol{j}}^{\boldsymbol{i}} \right)=B \right) or \left( \mathbb{K}\left( \boldsymbol{T}_{\boldsymbol{j}}^{\boldsymbol{i}} \right)=2 and \hat{\mathbb{K}}\left( \boldsymbol{T}_{\boldsymbol{j}}^{\boldsymbol{i}} \right)=A \right) \right\}}{p_{i}} \end{aligned} \right\}$$

where $I\{\}$ is an indicator function. This definition of $c_{k}$ allows us to compute the clustering accuracy based on the most likely cluster correspondence established by the clustering.

**Supplementary Materials Appendix C**: Members of the Nephrotic Syndrome Study Network (NEPTUNE)

**NEPTUNE Collaborating Sites**

*Atrium Health Levine Children’s Hospital, Charlotte, SC*: Susan Massengill^*^, Layla Lo^#^

*Cleveland Clinic, Cleveland, OH*: Katherine Dell^*^, John O’Toole^*^, John Sedor^**^, Victoria Grange^#^

*Children’s Hospital, Los Angeles, CA*: Ian Macumber^*^, Alyssa Parry^#^

*Children’s Mercy Hospital, Kansas City, MO*: Tarak Srivastava^*^, Kelsey Markus^#^

*Cohen Children’s Hospital, New Hyde Park, NY*: Christine Sethna^*^, Suzanne Vento^#^

*Columbia University, New York, NY:* Pietro Canetta^*^

*Duke University Medical Center, Durham, NC:* Opeyemi Olabisi^*^, Rasheed Gbadegesin^**^, Maurice Smith^#^

*Emory University, Atlanta, GA:* Laurence Greenbaum^*^, Chia-shi Wang^*^, Emily Yun^#^

*The Lundquist Institute, Torrance, CA:* Sharon Adler^*^, Janine LaPage^#^

*John H Stroger Cook County Hospital, Chicago, IL:* Amatur Amarah^*^

*Johns Hopkins Medicine, Baltimore, MD:* Meredith Atkinson^*^, Sara Boynton^#^

*Mayo Clinic, Rochester, MN:* John Lieske, Marie Hogan, Fernando Fervenza

*Medical University of South Carolina, Charleston, SC:* David Selewski^*^, Cheryl Alston^#^

*Montefiore Medical Center, Bronx, NY:* Kim Reidy^*^, Michael Ross^*^, Frederick Kaskel^**^, Patricia Flynn^#^

*New York University Medical Center, New York, NY:* Laura Malaga-Dieguez^*^, Olga Zhdanova^**^, Laura Jane Pehrson^#^, Melanie Miranda^#^

*The Ohio State University College of Medicine, Columbus, OH*: Salem Almaani^*^, Laci Roberts^#^

*Stanford University, Stanford, CA:* Richard Lafayette^*^, Shiktij Dave^#^

*Temple University, Philadelphia, PA:* Iris Lee^**^

*Texas Children’s Hospital at Baylor College of Medicine, Houston, TX*: Shweta Shah^*^, Sadaf Batla^#^ ^#^

*University Health Network Toronto:* Heather Reich^*^, Michelle Hladunewich^**^, Paul Ling^#^, Martin Romano^#^

*University of California at San Francisco, San Francisco, CA*: Paul Brakeman^*^, Daniel Schrader

*University of Colorado Anschutz Medical Campus, Aurora, CO*: James Dylewski^*^ Nathan Rogers^#^

*University of Kansas Medical Center, Kansas City, KS*: Ellen McCarthy^*^, Catherine Creed^#^

*University of Miami, Miami, FL:* Alessia Fornoni^*^, Miguel Bandes^#^

*University of Michigan, Ann Arbor, MI:* Matthias Kretzler^*^, Laura Mariani^*^, Zubin Modi^*^, A Williams^#^, Roxy Ni^#^

*University of Minnesota, Minneapolis, MN:* Patrick Nachman^*^, Michelle Rheault^*^, Amy Hanson^#^, Nicolas Rauwolf^#^

*University of North Carolina, Chapel Hill, NC:* Vimal Derebail^*^, Keisha Gibson^*^, Anne Froment^#^, Mary Mac McGown Collie^#^

*University of Pennsylvania, Philadelphia, PA:* Lawrence Holzman^*^, Kevin Meyers^**^, Krishna Kallem^#^, Aliya Edwards^#^

*University of Texas San Antonio, San Antonio, TX*: Samin Sharma^**^

*University of Texas Southwestern, Dallas, TX:* Elizabeth Roehm^*^, Kamalanathan Sambandam^**^, Elizabeth Brown^**^, Jamie Hellewege

*University of Washington, Seattle, WA:* Ashley Jefferson^*^, Sangeeta Hingorani^**^, Katherine Tuttle^**§^, Linda Manahan ^#^, Emily Pao^#^, Kelli Kuykendall^§^

*Wake Forest University Baptist Health, Winston-Salem, NC:* Jen Jar Lin^**^

*Washington University in St. Louis, St. Louis, MO*: Vikas Dharnidharka^*^

**Data Analysis and Coordinating Center:** *University of Michigan:* Matthias Kretzler^*^, Brenda Gillespie^**^, Laura Mariani^**^, Zubin Modi^**^, Eloise Salmon^**^, Howard Trachtman^**^, Tina Mainieri, Gabrielle Alter, Michael Arbit, Hailey Desmond, Sean Eddy, Damian Fermin, Wenjun Ju, Maria Larkina, Chrysta Lienczewski, Rebecca Scherr, Jonathan Troost, Amanda Williams, Yan Zhai; *Arbor Collaborative for Health:* Colleen Kincaid, Shengqian Li, Shannon Li; *Cleveland Clinic:* Crystal Gadegbeku^**^, *Duke University:* Laura Barisoni^**^; John Sedor^**^, *Harvard University:* Matthew G Sampson^**^; *Northwestern University:* Abigail Smith^**^; *University of Pennsylvania:* Lawrence Holzman^**^, Jarcy Zee^**^

**Digital Pathology Committee:** Carmen Avila-Casado *(University Health Network)*, Serena Bagnasco *(Johns Hopkins University)*, Lihong Bu *(Mayo Clinic)*, Shelley Caltharp *(Emory University)*, Clarissa Cassol *(Arkana)*, Dawit Demeke *(University of Michigan)*, Brenda Gillespie *(University of Michigan)*, Jared Hassler *(Temple University)*, Leal Herlitz *(Cleveland Clinic)*, Stephen Hewitt *(National Cancer Institute)*, Jeff Hodgin *(University of Michigan)*, Danni Holanda *(Arkana)*, Neeraja Kambham *(Stanford University)*, Kevin Lemley, Laura Mariani *(University of Michigan)*, Nidia Messias *(Washington University)*, Alexei Mikhailov *(Wake Forest)*, Vanessa Moreno *(University of North Carolina)*, Behzad Najafian *(University of Washington)*, Matthew Palmer *(University of Pennsylvania)*, Avi Rosenberg *(Johns Hopkins University)*, Virginie Royal *(University of Montreal)*, Miroslav Sekulik *(Columbia University)*, Barry Stokes *(Columbia University)*, David Thomas *(Duke University)*, Ming Wu *(University of New York)*, Michifumi Yamashita *(Cedar Sinai)*, Hong Yin *(Emory University)*, Jarcy Zee *(University of Pennsylvania)*, Yiqin Zuo *(University of Miami)*. Co-Chairs: Laura Barisoni *(Duke University)*, Cynthia Nast *(Cedar Sinai)*.

**Supplementary Materials Appendix D**: Members of the Cure Glomerulonephropathy (CureGN)

**CureGN Collaborators**

The CureGN Consortium members listed below, from within the four Participating Clinical Center networks and Data Coordinating Center, are acknowledged by the authors as Collaborators.

**CureGN Principal Investigators; *CureGN Site Principal Investigators; ^#^CureGN Lead Coordinators.

**CureGN Participating Clinical Centers (PCC) through Columbia University:**

*Columbia University, New York, NY, US*: Wooin Ahn, Gerald Appel, Paul Appelbaum, Revekka Babayev, Andrew Bomback, Pietro Canetta, Brenda Chan, Vivette Denise D'Agati, Samitri Dogra, Hilda Fernandez, Ali Gharavi^**^, William Hines, Syed Ali Husain, Namrata Jain, Krzysztof Kiryluk, Fangming Lin, Maddalena Marasa^#^, Glen Markowitz, Hila Milo Rasouly, Sumit Mohan, Nicola Mongera, Jordan Nestor, Thomas Nickolas, Jai Radhakrishnan, Maya Rao, Simone Sanna-Cherchi, Shayan Shirazian, Michael Barry Stokes, Natalie Uy, Anthony Valeri, Natalie Vena

*University of Warsaw, Warszawa, Poland:* Bartosz Foroncewicz, Barbara Moszczuk, Krzysztof Mucha*, Agnieszka Perkowska-Ptasińska

*Gaslini Children’s Hospital, Genoa, Italy:* Gian Marco Ghiggeri*, Francesca Lugani

**CureGN Participating Clinical Centers (PCC) through the Pediatric Nephrology Research Consortium:**

*Arkana Laboratories, Little Rock, AR, USA*: Josephine Ambruzs, Helen Liapis

*Children’s Hospital of Michigan, Detroit, MI, USA*: Rossana Baracco, Amrish Jain*

*Children’s Hospital of New Orleans/ LSU Health, New Orleans, LA, USA*: Isa Ashoor, Diego Aviles*

*Children’s Mercy Hospital, Kansas City, MO, USA*: Tarak Srivastava*

*Children’s National Medical Center, Washington DC, USA*: Sun-Young Ahn*

*Cincinnati Children’s Hospital Cincinnati, OH, USA*: Prasad Devarajan, Elif Erkan*, Donna Claes, Hillarey Stone

*Connecticut Children’s Medical Center, Hartford, CT, USA*: Sherene Mason*

*Duke Children’s Hospital Medical Center, Durham, NC, USA*: Rasheed Gbadegesin*

*East Carolina University Brody School of Medicine, Greenville, NC, USA*: Liliana Gomez-Mendez*

*Emory University, Atlanta, GA, USA*: Larry Greenbaum**, Chia-shi Wang, Hong (Julie) Yin

*Helen DeVos Children’s Hospital, Grand Rapids, MI, USA*: Yi Cai*, Goebel Jens, Julia Steinke

*Levine Children’s Hospital/Atrium Health, Charlotte, NC, USA*: Donald Weaver*

*Lurie Children’s Hospital, Chicago IL, USA*: Jerome Lane*

*Mayo Clinic, Rochester, MN, USA*: Carl Cramer*

*Medical College of Wisconsin, Milwaukee, WI, USA*: Cindy Pan, Neil Paloian, Rajasree Sreedharan*

*Medical University of South Carolina, Charleston SC, USA*: David Selewski, Katherine Twombley*

*Nationwide Children’s Hospital, Columbus, OH, USA*: Corinna Bowers^#^, Mary Dreher^#^ Mahmoud Kallash*, John Mahan, Samantha Sharpe^#^, William Smoyer**

*Oregon Health and Science University, Portland, OR, USA*: Amira Al-Uzri*, Sandra Iragorri

*Riley Children’s Hospital, Indianapolis, IN, USA*: Myda Khalid*

*Cardinal Glennon Children’s Medical Center/ St. Louis University, St. Louis, MO, USA*: Craig Belsha*

*Texas Children’s Hospital, Houston, TX, USA*: Joseph Alge*, Michael Braun, AC Gomez, Scott Wenderfer*

*Texas Tech Health Sciences Center, Amarillo, TX, USA*: Tetyana Vasylyeva*

*Children’s of Alabama, University of Alabama, Birmingham, AL, USA*: Daniel Feig*

*University of Colorado Children’s Hospital, Colorado, Aurora, CO, USA*: Gabriel Cara Fuentes, Melisha Hannah*

*University of Iowa Children’s Hospital, Iowa City, IA, USA*: Carla Nester*

*University of Kentucky, Lexington, KY, USA*: Aftab Chishti*

*University of Louisville, Louisville, KY, USA*: Jon Klein^**^

*Holtz Medical Center, University of Miami, Miami, FL, USA*: Chryso Katsoufis, Wacharee Seeherunvong*

*University of Minnesota Children’s Hospital, Minneapolis, MN, USA*: Michelle Rheault*

*University of New Mexico Health Sciences Center, Albuquerque, NM, USA*: Craig Wong*

*University of Oklahoma Health Sciences Center, Oklahoma City, OK, USA*: Nisha Mathews*

*University of Virginia, Charlottesville, VA, USA*: John Barcia*, Agnes Swiatecka-Urban

*University of Wisconsin, Madison, WI, USA*: Sharon Bartosh*

*Vanderbilt Children’s Hospital, Nashville TN, USA*: Tracy Hunley*

*Washington University in St. Louis, St. Louis, MO, USA*: Vikas Dharnidharka*, Joseph, Gaut

**CureGN Participating Clinical Centers (PCC) through the University of North Carolina:**

*Hôpital Maisonneuve-Rosemont, Montreal, Canada*: Louis-Philippe Laurin*, Virginie Royal

*Medical University of South Carolina, Charleston, SC, USA*: Anand Achanti, Milos Budisavljevic*, Sally Self

*Northwestern University, Chicago, IL, USA*: Cybele Ghossein, Yonatan Peleg, Shikha Wadhwani*

*Ohio State University, Columbus, OH, USA*: Salem Almaani, Isabelle Ayoub, Tibor Nadasdy, Samir, Parikh, Brad Rovin*

*University of Chicago, Chicago, IL, USA*: Anthony Chang

*University of Alabama at Birmingham, Birmingham, AL, USA*: Huma Fatima, Bruce Julian, Jan Novak, Matthew Renfrow, Dana Rizk*

*University of North Carolina Kidney Center, Chapel Hill, NC, USA*: Dhruti Chen, Vimal Derebail, Ronald Falk**, Keisha Gibson, Dorey Glenn, Susan Hogan, Koyal Jain, J. Charles Jennette, Amy Mottl*, Caroline Poulton^#^, Manish Kanti Saha

*Vanderbilt University, Nashville, TN, USA*: Agnes Fogo, Neil Sanghani*

*Virginia Commonwealth University, Richmond, VA, USA*: Jason Kidd*, Selvaraj Muthusamy

**CureGN Participating Clinical Centers (PCC) through the University of Pennsylvania:**

*MetroHealth Medical Center/Case Western Reserve University, Cleveland, OH, USA*: Jeffrey Schelling*

*Cedars-Sinai Health System, Los Angeles, CA, USA*: Jean Hou

*Children’s Hospital of LA, Los Angeles, CA, USA*: Kevin Lemley*, Warren Mika, Pierre Russo

*Children’s Hospital of Philadelphia, Philadelphia, PA, USA*: Michelle Denburg, Amy Kogon, Kevin Meyers*, Madhura Pradhan

*Cleveland Clinic, Cleveland, OH, CA*: Raed Bou Matar*, John O'Toole*, John Sedor*

Cohen Children’s Medical Center, New Hyde Park, NY, USA: Christine Sethna*, Suzanne Vento ^#^

*Johns Hopkins University, Baltimore, MD, USA*: Mohamed Atta, Serena Bagnasco, Alicia Neu, John Sperati*

*Lundquist Institute at Harbor-UCLA Medical Center, Torrance, CA, USA*: Sharon Adler*, Tiane Dai, Ram Dukkipati

*Mayo Clinic, Rochester, MN, USA*: Fernando Fervenza*, Sanjeev Sethi

*Montefiore Medical Center, The Bronx, New York, NY, USA*: Frederick Kaskel, Kaye Brathwaite, Kimberly Reidy*

*New York University, New York, NY, USA*: Joseph Weisstuch, Ming Wu, Olga Zhdanova

*NIDDK, Bethesda, MD, USA*: Jurgen Heymann, Jeffrey Kopp*, Meryl Waldman, Cheryl Winkler

*Spokane Providence Medical Center, Spokane, WA, USA*: Katherine Tuttle*

*Stanford University, Palo Alto, CA, USA*: Jill Krissberg, Richard Lafayette*, Kamal Fahmeedah, Elizabeth Talley

*Sunnybrook Health Sciences Centre, Toronto, Canada*: Michelle Hladunewich*

*The Hospital for Sick Children, Toronto, Canada*: Rulan Parekh*

*University Health Network, Toronto, Canada*: Carmen Avila-Casado, Daniel Cattran*, Reich Heather, Philip Boll

*University of Miami, Miami, FL, USA*: Yelena Drexler, Alessia Fornoni*

*University of Michigan, Ann Arbor, MI, USA*: Brooke Blazius*, Jeffrey Hodgin, Andrea Oliverio

*University of Pennsylvania, Philadelphia, PA, USA*: Jon Hogan, Lawrence Holzman**, Matthew Palmer, Gaia Coppock

*University of Pittsburgh School of Medicine, Pittsburgh, PA, USA*: Blaise Abromovitz*, Michael Mortiz*

*University of Washington, Seattle, WA, USA*: Charles Alpers, J. Ashley Jefferson*

*UT Southwestern, Dallas, TX, USA*: Elizabeth Brown, Kamal Sambandam*, Bethany Roehm

**Data Coordinating Center (DCC):**

*Arbor Research Collaborative for Health, Ann Arbor, MI, USA*: John Graff, Abigail Smith

*Cedar Sinai Medical Center, Los Angeles, CA, USA*: Cynthia Nast

*Duke University, Durham, NC, USA*: Laura Barisoni

*University of Michigan, Ann Arbor, MI, USA*: Brenda Gillespie**, Bruce Robinson**, Matthias Kretzler, Laura Mariani**

**Steering Committee Chair:** Lisa M. Guay-Woodford, Children’s Hospital of Pennsylvania, Philadelphia, PA, USA

**Supplementary Materials Table 1**: GMM-based and K-means clustering accuracy for increasing resampling variability $\sigma_{R}^{2}$

| $\sigma_{R}^{2}$ | Clustering accuracy GMM (%) | Clustering accuracy  K-means (%) |
| --- | --- | --- |
| 1 | 99.8 | 99.5 |
| 6 | 95.6 | 95.2 |
| 11 | 90.7 | 90.5 |
| 16 | 86.8 | 86.8 |
| 21 | 83.5 | 83.8 |
| 26 | 80.7 | 81.4 |
| 31 | 78.3 | 79.5 |
| 36 | 76.6 | 77.9 |
| 41 | 75.2 | 76.5 |
| 46 | 73.8 | 75.3 |

*Clustering accuracies* $c_{k}$ *for GMM-based clustering and K-means described in Section 4b for the simulation study results depicted in Figure 5 and Supplementary Materials Figure 2.*

**Supplementary Materials Table 2**: Average greatest proportion of simulated tubules classified into a cluster by GMM-based clustering for one underlying tubular cluster as resampling variability $\sigma_{R}^{2}$ increases

| $\sigma_{R}^{2}$ | Greatest proportion of tubules classified into a cluster (%) |
| --- | --- |
| 1 | 61.7 |
| 6 | 81.8 |
| 11 | 83.3 |
| 16 | 84.0 |
| 21 | 83.1 |
| 26 | 84.7 |
| 31 | 84.1 |
| 36 | 83.3 |
| 41 | 83.3 |
| 46 | 83.2 |

*Mean (across simulation repetitions) greatest proportion of tubules classified as belonging to a cluster identified by GMM-based clustering when there was one underlying tubular cluster, as described in Section 4a. For each simulation repetition for a given simulation setting (*$\sigma_{R}^{2}$ *value with* $q=10$*,* $s_{\beta}^{*}=0.8$*, and* $n=500$*), the greatest proportion of tubules classified in a cluster by GMM-based clustering is recorded. Then, we take the mean of these greatest proportions across simulation repetitions to get the mean greatest proportion of tubules classified into a cluster for that simulation setting.*

**Supplementary Materials Table 3**: List of all tubular image features for NEPUTNE and CureGN data analysis

| Feature |
| --- |
| Tubular basement membrane area |
| Tubular epithelium + lumen area |
| Tubule area |
| Lumen area |
| Tubular epithelium area |
| Nuclei area |
| Tubule diameter |
| Lumen diameter |
| Tubular epithelium + lumen diameter |
| Tubular basement membrane average thickness |
| Tubular epithelium average thickness |
| Tubule average thickness |
| Lumen average thickness |
| Tubular epithelium + lumen average thickness |
| Lumen smoothness |
| Tubular basement membrane smoothness |
| Tubular epithelium + lumen smoothness |
| Tubular epithelium + lumen area to tubular basement membrane area ratio |
| Tubular epithelium area to tubular basement membrane area ratio |
| Lumen area to tubular basement membrane area ratio |
| Tubule area to tubular basement membrane area ratio |
| Tubular epithelium + lumen area to tubule area ratio |
| Lumen + tubular epithelium area to lumen area ratio |
| Tubular epithelium area to tubular epithelium + lumen area ratio |
| Lumen area to tubule area ratio |
| Tubular epithelium area to tubule area ratio |
| Lumen area to tubular epithelium area ratio |
| Lumen diameter to tubular basement membrane thickness ratio |
| Tubule diameter to tubular basement membrane thickness ratio |
| Tubular epithelium + lumen diameter to tubular basement membrane thickness ratio |
| Tubular epithelium thickness to tubular basement membrane thickness ratio |
| Lumen diameter to tubule diameter ratio |
| Lumen diameter to tubular epithelium + lumen diameter ratio |
| Lumen diameter to tubular epithelium thickness ratio |
| Tubule diameter to tubular epithelium + lumen diameter ratio |
| Tubular epithelium thickness to tubule diameter ratio |
| Tubular epithelium thickness to tubular epithelium + lumen diameter ratio |
| Tubular basement membrane diameter to tubule diameter ratio |
| Lumen diameter to tubular basement membrane diameter ratio |
| Tubular basement membrane diameter to tubular epithelium + lumen diameter ratio |
| Tubular epithelium thickness to tubular basement membrane diameter ratio |
| Tubular epithelium diameter to tubular epithelium + lumen diameter ratio |
| Tubular epithelium diameter to tubule diameter ratio |
| Lumen diameter to tubular epithelium diameter ratio |
| Tubular epithelium diameter to tubular basement membrane diameter ratio |
| Tubular epithelium diameter to tubular basement membrane thickness ratio |
| Nuclei area to tubular epithelium area ratio |
| Nuclei area to tubular epithelium + lumen area ratio |
| Nuclei area to tubule area ratio |
| Lumen area to nuclei area ratio |
| Nuclei area to tubular basement membrane area ratio |
| Nuclei centroid to lumen border average distance |
| Nuclei centroid to tubular basement membrane border average distance |
| Nuclei border to lumen border average distance |
| Nuclei border to tubular basement membrane border average distance |
| Nuclei border to tubular epithelium border average distance |
| Nuclei centroid to tubular epithelium border average distance |

*List of all tubular image features used by the naïve approach and CLUSSO to predict kidney function for analysis of subjects in the NEPTUNE and CureGN cohorts described in Section 5.*

**Supplementary Materials Figure 1**: Simulation study results with correlated tubules


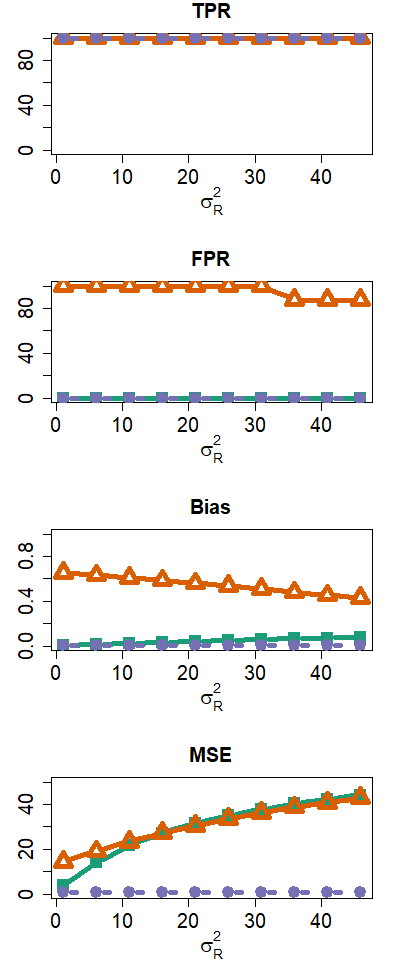


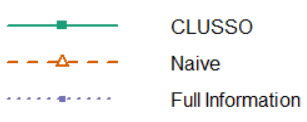


*Simulation study results for applying CLUSSO with GMM-based clustering, naïve, and Full Information Structured Lasso methods when the tubules were generated from a multivariate normal distribution in which tubules from different clusters had different means, tubules had a correlation of 0.5 with each other, and the image features were independent. A description of generating observed feature matrices using this matrix normal distribution is described in Section 4a. For these simulations,* $q=10$ *is the number of features,* $s_{\beta}^{*}=0.8$ *is the proportion of* $\boldsymbol{\beta}^{\boldsymbol{*}}$*coefficients which are zero,* $n=500$ *is the sample size, and* $\sigma_{R}^{2}\in\{1,6,11,16,21,26,31,36,41,46\}$ *is the resampling variability. All results are averaged by taking the median across the 1000 simulation repetitions.*

**Supplementary Materials Figure 2**: Simulation study results with three underlying tubular clusters from varying resampling variability $\sigma_{R}^{2}$


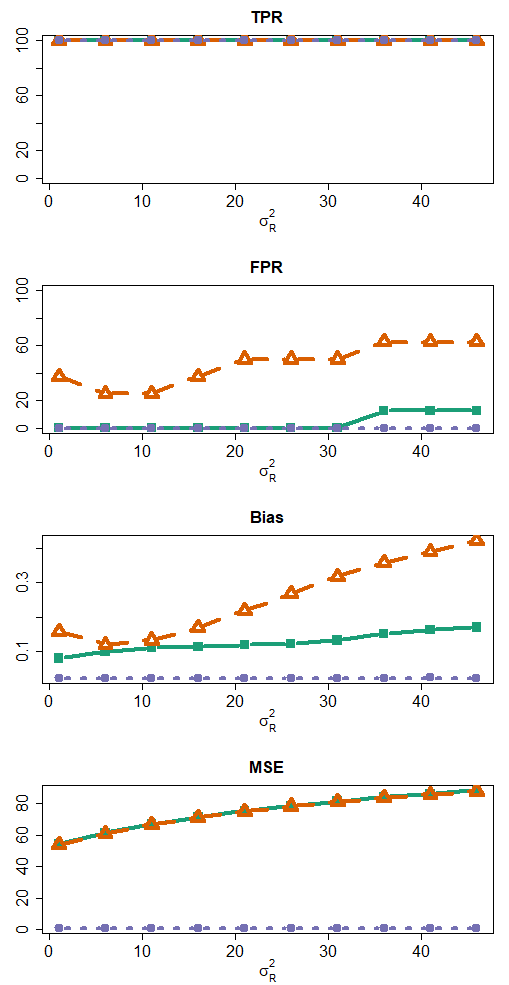


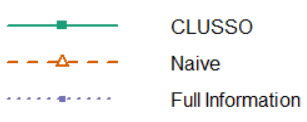


*Simulation study results for applying CLUSSO with GMM-based clustering, naïve, and Full Information Structured Lasso methods for three underlying tubular clusters. GMM-based clustering was used to classify tubules into two clusters, even though the true number of latent tubular clusters was three. A description of generating observed feature matrices from the three latent tubular clusters is described in Section 4a. For these simulations,* $q=10$ *is the number of features,* $s_{\beta}^{*}=0.8$ *is the proportion of* $\boldsymbol{\beta}^{\boldsymbol{*}}$*coefficients which are zero,* $n=500$ *is the sample size, and* $\sigma_{R}^{2}\in\{1,6,11,16,21,26,31,36,41,46\}$ *is the resampling variability. All results are averaged by taking the median across the 1000 simulation repetitions.*

**Supplementary Materials Figure 3**: Confusion matrices for GMM-based clustering and K-means clustering under resampling variability $\sigma_{R}^{2}=46$ for one simulation repetition


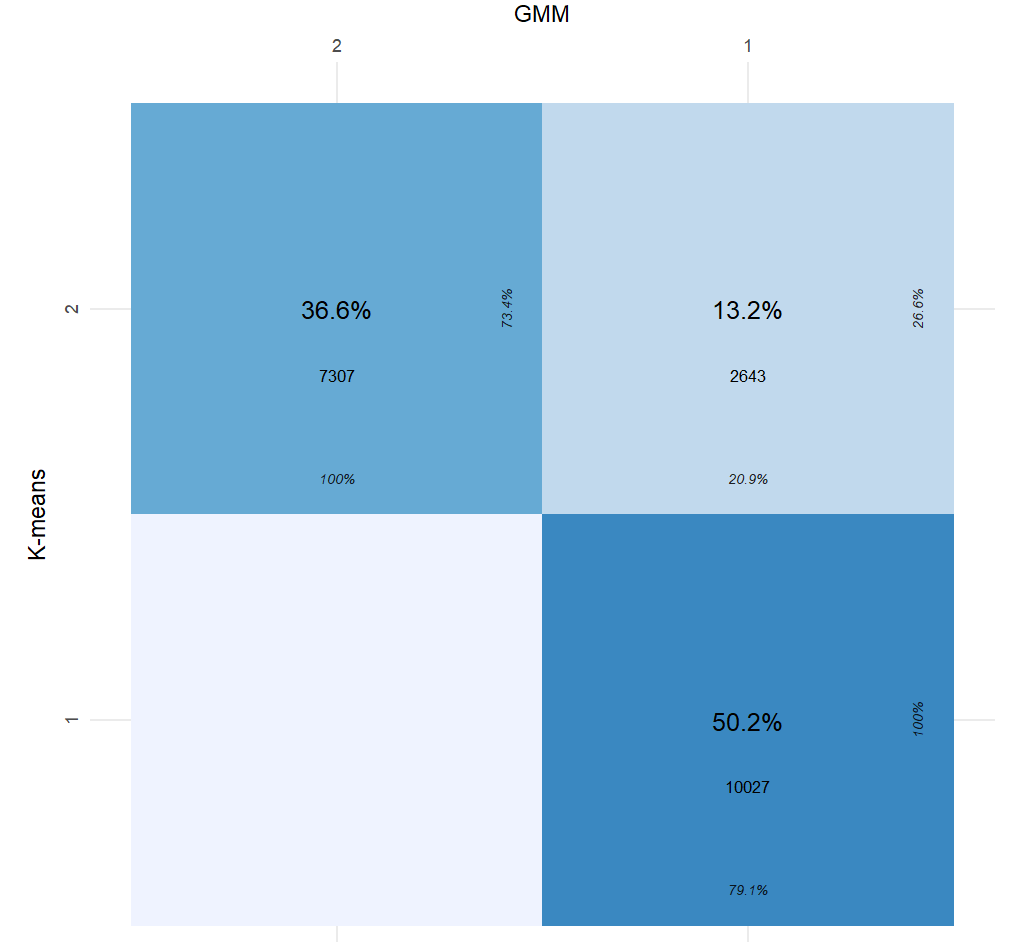


*Confusion matrices for GMM-based clustering and K-means clustering for one simulation repetition when* $\sigma_{R}^{2}=46$*,* $q=10$*,* $s_{\beta}^{*}=0.8$*, and* $n=500$. *This confusion matrix compares the clustering label assignments of these two algorithms when tubules are classified into one of two different clusters. We see that these two clustering labels have a correspondence of 86.8%.*

**Supplementary Materials Figure 4**: CLUSSO with K-means simulation study results from varying resampling variability $\sigma_{R}^{2}$


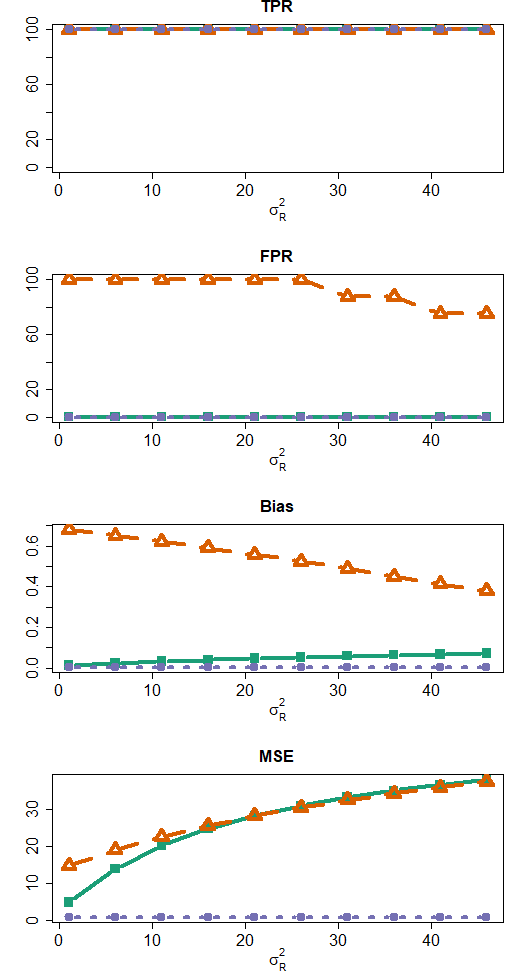


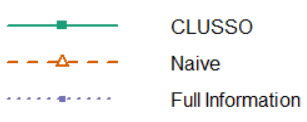


*Simulation study results for applying CLUSSO with the K-means algorithm, naïve, and Full Information Structured Lasso methods. For these simulations where* $q=10$ *is the number of features,* $s_{\beta}^{*}=0.8$ *is the proportion of* $\boldsymbol{\beta}^{\boldsymbol{*}}$*coefficients which are zero,* $n=500$ *is the sample size, and* $\sigma_{R}^{2}\in\{1,6,11,16,21,26,31,36,41,46\}$ *is the resampling variability. All results are averaged by taking the median across the 1000 simulation repetitions.* **Supplementary Materials Figure 5**: Distributions of number of tubules per subject from NEPTUNE and CureGN data.

*
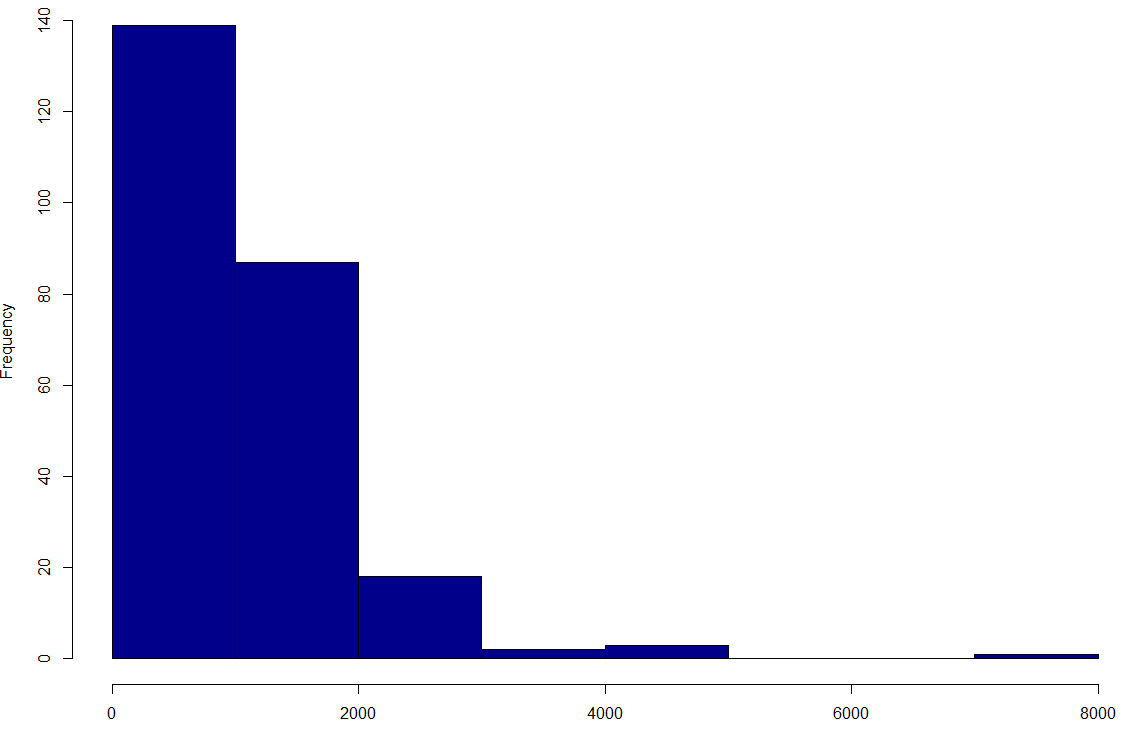
*

*
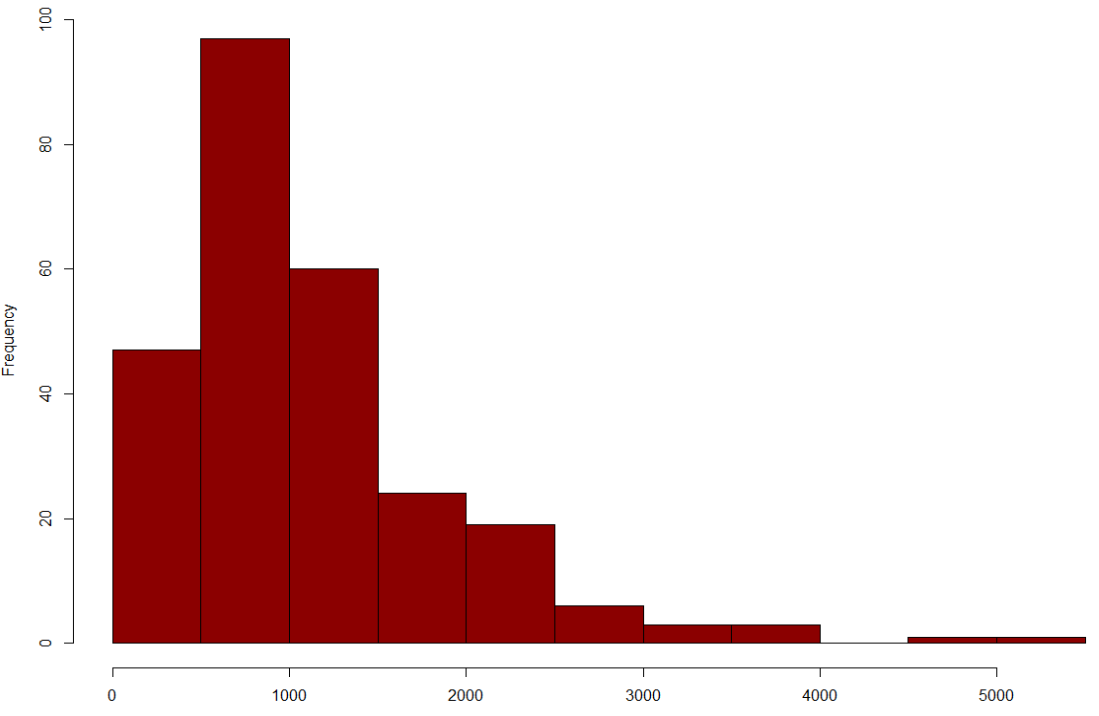
*

*Histograms of the number of tubules per subject for NEPTUNE cohort (blue) and CureGN cohort (red) described in Section 5.*
